# Single-Nucleus Transcriptomics Reveals Glial Metabolic–Immune Rewiring and Intercellular Signaling Disruption in Chronic Migraine

**DOI:** 10.3390/biom15070942

**Published:** 2025-06-28

**Authors:** Shuangyuan Hu, Zili Tang, Shiqi Sun, Lu Liu, Yuyan Wang, Longyao Xu, Jing Yuan, Ying Chen, Mingsheng Sun, Ling Zhao

**Affiliations:** 1 School of Acupuncture and Tuina, Chengdu University of Traditional Chinese Medicine, Chengdu 611137, China; doublehu@stu.cdutcm.edu.cn (S.H.); tangzili@cdutcm.edu.cn (Z.T.); shiqisun777@gmail.com (S.S.); liulu@stu.cduecm.edu.cn (L.L.); wangyuyan@stu.cdutcm.edu.cn (Y.W.); xulongyao54@gmail.com (L.X.); yuanjing@cdutcm.edu.cn (J.Y.); chenying@stu.cdutcm.edu.cn (Y.C.); 2 Key Laboratory of Acupuncture for Senile Disease, Chengdu University of Traditional Chinese Medicine, Ministry of Education, Chengdu 611137, China; 3 Acupuncture & Chronobiology Key Laboratory of Sichuan Province, Chengdu 611137, China

**Keywords:** chronic migraine, single-nucleus transcriptomics, trigeminal nucleus caudalis, astrocytes, microglia, glial heterogeneity, pseudotime trajectory, ligand–receptor signaling

## Abstract

Chronic migraine (CM) is a debilitating neurological disorder, yet the glial-specific mechanisms underlying its pathophysiology in the trigeminal nucleus caudalis (TNC)—a critical hub for craniofacial pain processing—remain poorly understood. Here, we employed single-nucleus RNA sequencing (snRNA-seq) to resolve cell-type-specific transcriptional landscapes in a nitroglycerin (NTG)-induced CM rat model, with a particular focus on microglia and astrocytes. We identified 19 transcriptional clusters representing nine major cell types, among which reactive microglia (NTG-Mic) and astrocytes (NTG-Asts) were markedly expanded. The NTG-Mic displayed a glycolysis-dominant, complement-enriched state, whereas the NTG-Asts exhibited concurrent activation of amino acid transport and cytokine signaling pathways. Pseudotime trajectory analysis revealed bifurcated glial activation paths, with NTG driving both cell types toward terminal reactive states. Intercellular communication inference uncovered suppressed homeostatic interactions (e.g., CSF1-CSF1R) alongside enhanced proinflammatory signaling (e.g., FGF1-FGFR2, PTN-SDC4), particularly affecting neuron–glia and glia–glia crosstalk. Together, these findings define a high-resolution atlas of glial reprogramming in CM, implicating state-specific metabolic–immune transitions and dysregulated glial communication as potential targets for therapeutic intervention.

## 1. Introduction

Chronic migraine (CM) is a disabling neurological disorder affecting over 2% of adults globally, with substantial clinical and socioeconomic burdens due to its high attack frequency, severity, and limited treatment responsiveness [1]. Despite advances in therapy, 40–50% of patients remain refractory to first-line treatments [2], highlighting the unmet need to define the mechanisms driving migraine chronification.

Genetic susceptibility [3] and autoimmune-mediated brain alterations [4] represent critical upstream etiologies in CM. However, converging evidence suggests that, regardless of the initiating cause, CM is ultimately characterized by the sustained activation of neuroinflammation and neuroimmune and glial pathways within the trigeminovascular system. The trigeminal nucleus caudalis (TNC) is a central hub for nociceptive integration and a putative site of persistent neuroinflammation in CM [5]. While the TNC comprises diverse central nervous system (CNS) cell types (e.g., neurons, microglia, astrocytes), its role in pain transmission hinges on specialized synaptic connectivity between trigeminal afferents and second-order neurons [6,7]. Within this milieu, microglia and astrocytes are key mediators: the microglia initiate inflammatory cascades via complement activation and cytokine release [8,9], while the astrocytes exacerbate central sensitization by disrupting glutamate homeostasis and promoting gliosis [10,11]. However, conventional bulk-tissue analyses have masked cell-type-specific dynamics, leaving critical gaps in understanding which glial subpopulations drive CM, how metabolic–immune crosstalk shapes their reactivity, and which neuron–glia signaling pathways sustain chronic pain.

Recent advances in single-nucleus RNA sequencing (snRNA-seq) have resolved glial heterogeneity in neurodegenerative [12] and neuropathic pain contexts [13,14,15], yet the TNC—the brainstem nexus of migraine pathogenesis—remains uncharacterized at the single-cell resolution. Although Yang et al. profiled the trigeminal ganglion in both human and mouse migraine models [16], glial adaptations within the TNC, where central sensitization is orchestrated, are unexplored.

Here, we hypothesized that chronic nitroglycerin (NTG) exposure induces subtype-specific glia reprogramming in the TNC, coupling metabolic remodeling with immune activation and maladaptive neuron–glia signaling. We used a validated chronic NTG rat model that reproduces key clinical features of CM, including trigeminal activation, hyperalgesia, and molecular overlap with human migraine transcriptomes [17,18]. While this model does not recapitulate the genetic or autoimmune origins of migraine, it robustly induces inflammatory and sensitization mechanisms, making it a widely accepted platform for dissecting downstream glial and neuroimmune responses relevant to CM. Using this model, we leveraged snRNA-seq to achieve unbiased, high-resolution profiling of transcriptional states across complex tissues, enabling us to decode microglial and astrocytic states, trajectory shifts, and intercellular communication networks. Our findings reveal a glia-centric framework for CM pathogenesis and nominate novel targets for therapeutic intervention.

## 2. Materials and Methods

### 2.1. Establishment of a CM Rat Model

To closely mimic the pathological state of human chronic migraine (CM) and avoid hormonal confounding, twelve male Sprague-Dawley (SD) rats (200 ± 20 g; Ensiweier Animal Company, Chengdu, China; SCXK(Xiang)2019-0004; 430727200101073785) were used in this study. The rats were housed under controlled conditions (temperature: 25 ± 2 °C; humidity: 50 ± 10%; 12 h light/dark cycle). Following a 7-day acclimatization period, the rats were randomly divided into two groups (n = 6/group): control (CON) and nitroglycerin-induced CM model (NTG). The NTG group received subcutaneous injections of nitroglycerin (NTG, 10 mg/kg; Yimin Pharmaceutical Co., Ltd., Beijing, China) on days 1, 3, 5, 7, and 9, while the CON group received equal volumes of saline [19].

All the procedures were approved by the Experimental Animal Ethics Committee of Chengdu University of Traditional Chinese Medicine (Approval No. 2019-23).

### 2.2. Assessment of Paw Withdrawal Mechanical Threshold (PWMT)

The mechanical pain threshold was evaluated using Von Frey filaments applied vertically to the plantar surface of the hind paw. A positive response was defined as paw withdrawal, contraction, lifting, or licking. Each rat was tested three times at 3 min intervals, both 2 h pre- and post-NTG injection. The average force required to elicit a response was recorded as the PWMT.

### 2.3. Assessment of Tail-Flick Test (TFT)

Thermal nociception was assessed using a radiant heat tail-flick analgesia instrument (Chengdu Techman Software Co., Ltd., Chengdu, China). The heat source was positioned 1–3 cm from the tail, with a cutoff time of 10 s to prevent tissue damage. Tail-flick latency was recorded across three trials per rat at 3 min intervals, both 2 h pre- and post-NTG injection. The average latency was calculated, and the experimenters were blinded to the group assignments.

### 2.4. Single-Cell Suspension Preparation and Nuclei Isolation

Trigeminal nucleus caudalis (TNC) tissues were collected from the CON and NTG groups. The tissues were mechanically dissociated with the Cell Nuclear Isolation Kit (Shbio, 52009–10, Shanghai, China) following the manufacturer’s protocol. Nuclei counts were determined using a cell counter (Thermo Fisher Scientific, Waltham, MA, USA). RNA barcoding and library preparation were performed with Chromium Single Cell 3′ Library (10X Genomics, Pleasanton, CA, USA) and Gel Bead Kit v3 (10X Genomics, Pleasanton, CA, USA) and sequenced on an Illumina NovaSeq 6000 platform (Illumina, San Diego, CA, USA).

### 2.5. snRNA-Seq Data Quality Control and Preprocessing

Raw sequencing data were processed using Cell Ranger (v3.0.1) with default settings. Reads were aligned to the Rattus norvegicus reference genome (Rnor_6.0) using STAR [20], and gene-barcode matrices were generated by counting unique molecular identifiers (UMIs). Cells with <500 or >4000 detected genes, >15% mitochondrial content, or extreme UMI/gene counts (>2 SD) were excluded. Doublets were removed using DoubletFinder (v2.0.3) [21], and contamination was mitigated with DecontX [22]. Data normalization and scaling were performed using Seurat [23], and clusters were visualized with Uniform Manifold Approximation and Projection (UMAP). Highly variable genes (n = 2000) were selected using the FindVariableFeatures function.

### 2.6. Cell Type Identification and Functional Annotation

Cluster-specific marker genes as well as differentially expressed genes (DEGs) across clusters were identified using the FindMarkers function (min.pct = 0.25), which filters out genes expressed in <25% of the cells. Cell identities were assigned based on the CellMarker database (CellMarker 2.0, http://www.bio-bigdata.center/index.html, accessed on 20 April 2025) [24]. The major cell types and their representative markers were as follows: neurons (Neus, *Syt1*, *Snap25*), oligodendrocytes (Olgs, *Mog, Tubb4a*), oligodendrocyte precursor cells (OPCs, *Pdgfra*, *Vcan*) [25], astrocytes (Asts, *Gfap*, *Aqp4*), microglia (Mic, *Cx3cr1*, *Tmem119*, *Sall1*), macrophages (Macs, *Mrc1*, *F13a1*), endothelial cells (Ends, *Flt1*, *Cyyr1*), and fibroblasts (Fibs, *Col1a1*, *Col3a1*). In addition, we annotated an intermediate progenitor population (IPCs), which transcriptionally bridged proliferative OPCs and terminally differentiated oligodendrocytes, consistent with previous single-cell studies [26].

Subpopulations of interest (e.g., microglia, astrocytes) were isolated using the SubsetData function, re-clustered using t-distributed stochastic neighbor embedding (t-SNE) and uniform manifold approximation and projection (UMAP), and annotated with canonical markers using the FindClusters function.

### 2.7. Estimating Cellular Composition Preference Using Ro/e

The ratio of observed to expected (Ro/e) was used to assess the enrichment or depletion of specific cell types relative to expected frequencies under a null model. Following Cheng et al. [27], we calculated Ro/e between groups to quantify the relative over- or underrepresentation of cell types using chi-squared tests (chisq.test in R). An Ro/e value > 1 indicates overrepresentation (enrichment), while Ro/e < 1 indicates underrepresentation (depletion). In our study, this approach enabled the quantitative assessment of cell population shifts between the control and NTG-treated groups, revealing specific glial subsets that were disproportionately expanded or contracted in CM conditions.

### 2.8. Cell-Type-Specific Inflammation Score Analysis

AUCell (v1.22.0) was used to compute inflammation scores per cell using the “HALLMARK INFLAMMATORY RESPONSE” gene set from msigdbr (v7.5.1). Cells with median AUCell scores >0 were designated as hyperinflammatory.

### 2.9. Pathway Enrichment Analysis

Gene set variation analysis (GSVA), Gene Ontology (GO), and the Kyoto Encyclopedia of Genes and Genomes (KEGG) pathway were performed using clusterProfiler (v4.8.3) with gene sets from the msigdbr (v7.5.1) database. Pathways with adjusted *p*-values < 0.05 were considered significantly enriched. The results were visualized using ggplot2 and pheatmap.

### 2.10. Pseudotime Trajectory Analysis

Monocle2 (v2.28.0) was used for trajectory analysis [28]. Seurat-derived raw count matrices were converted into CellDataSet objects using importCDS. Cell ordering genes (qval < 0.01) were selected with differentialGeneTest, and dimensionality reduction was performed using reduceDimension (method = “DDRTree”, max_components = 2). Cells were ordered using orderCells, and trajectories were visualized with plot_cell_trajectory. The expression of the selected genes along the pseudotime trajectory was visualized using plot_genes_in_pseudotime function.

### 2.11. Cell–Cell Communication Analysis

To enable ligand–receptor interaction analysis with CellChat [29], which is currently limited to human and mouse genomes, the rat genes were converted to their mouse orthologs using the homologene R package (v1.4.68.19.3.27). The count data were then imported, and default parameters were applied for identifyOverExpressedGenes and identifyOverExpressedInteractions. A precompiled mouse protein–protein interaction (PPI) network was incorporated for enhanced pathway inference based on Secreted Signaling Database. Core functions, including computeCommunProbPathway, computeCommunProb, and aggregateNet, were employed to estimate communication probabilities and construct aggregated signaling networks. Finally, differential intercellular interactions between groups were identified using compareInteractions, netVisual_diffInteraction, and netVisual_bubble.

### 2.12. Statistical Analysis

Statistical analyses were performed using GraphPad Prism (v10). PWMT and TFT data were analyzed using two-way repeated-measures Analysis of Variance (ANOVA), with subgroup and time as factors. Unpaired *t*-tests were used for pairwise comparisons. Unless otherwise stated, statistical significance was defined as *p* < 0.05. Data are presented as means ± SD.

## 3. Results

### 3.1. NTG Induces Progressive Hyperalgesia in a Rat Model of CM

**Figure 1:**
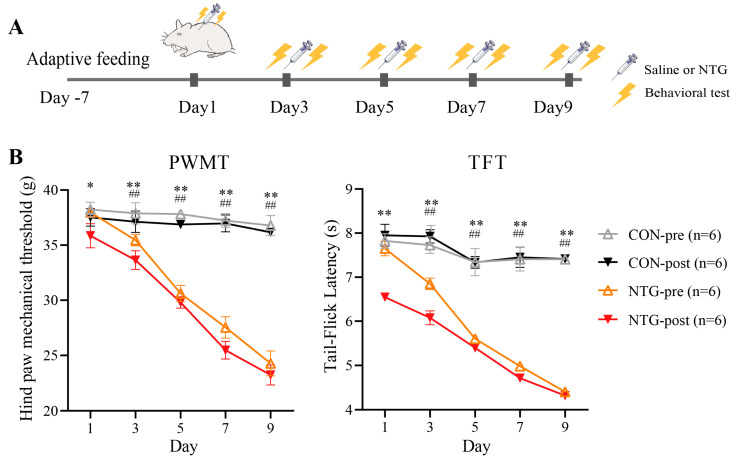
NTG-induced CM model in rats. (**A**) Schematic of the experimental timeline. Rats received NTG or saline (CON) injections, with behavioral tests conducted before (pre) and after (post) treatment. (**B**) Mechanical and thermal pain thresholds assessed by paw withdrawal mechanical threshold (PWMT) and tail-flick latency (TFT). Data presented as mean ± SD. ##: NTG-pre vs. CON-pre, *p* < 0.01; *: NTG-post vs. CON-post, *p* < 0.05; **: NTG-post vs. CON-post, *p* < 0.01.

To establish a CM model, rats received intermittent subcutaneous injections of NTG over nine days (Figure 1A). Mechanical and thermal sensitivity were assessed by using PWMT and TFT, measured two hours before (pre) and after (post) each injection.

At baseline (day 1, pre-injection), the PWMT and TFT values were comparable between NTG-treated and control (CON) rats. However, following NTG administration, both PWMT and TFT scores were significantly reduced in the NTG group compared with those in CON (NTG-post vs. CON-post, * *p* < 0.05; ** *p* < 0.01; Figure 1B), indicating acute hyperalgesia. This hypersensitivity intensified progressively with repeated dosing, peaking on day 9. Notably, even pre-injection thresholds on days 2–9 were significantly lower in NTG animals (^##^ *p* < 0.01), suggesting a transition to chronic pain. These behavioral data confirm the successful induction of a CM-like phenotype characterized by persistent mechanical and thermal hyperalgesia.

### 3.2. NTG Drives Cellular Remodeling and Neuroinflammatory Activation in the TNC

**Figure 2:**
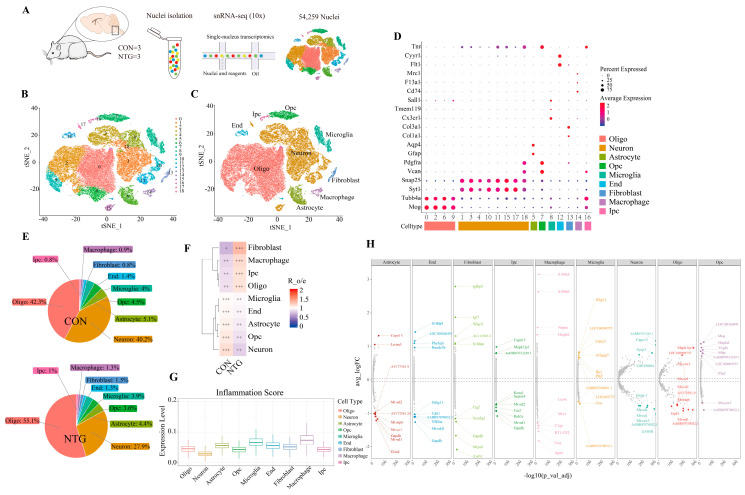
SnRNA-seq reveals altered cellular composition and glial activation in the TNC following NTG administration. (**A**) Schematic overview of the study design. TNC tissues were collected from 3 biological replicates per group using the 10x Genomics platform. (**B**) The t-SNE projection of 54,259 nuclei, colored by unsupervised clustering (19 distinct clusters). (**C**) The t-SNE plot re-annotated by major cell types (9 populations) after merging subclusters. (**D**) Bubble plot of cell-type-specific marker genes. Dot size reflects the percentage of cells expressing the gene; color indicates mean scaled expression. (**E**) Proportional shifts in cell type abundance between NTG and CON groups. Pie charts show normalized cell fractions (total = 1). (**F**) Heatmap of cell type proportion changes, quantified by Ro/e ratio. +++, Ro/e > 1; ++, 0.8 < Ro/e ≤ 1; +, 0.2 ≤ Ro/e ≤ 0.8. (**G**) Heatmap of top 10 DEGs across subpopulations (rows: genes; columns: clusters). (**H**) Inflammatory activity scores across cell types, derived from gene signatures.

To comprehensively characterize the NTG-induced changes in cell composition, we performed snRNA-seq on nuclei isolated from the TNC tissues of the CON and NTG rats. Following stringent quality control, 54,259 high-quality nuclei expressing 21,637 genes were retained for downstream analysis (Figure 2A). Unsupervised clustering and dimensionality reduction using t-SNE identified 19 transcriptionally distinct clusters (Figure 2B), which were subsequently consolidated into nine major cell types annotated by canonical markers (Figure 2C,D), including neurons (Neus), astrocytes (Asts), microglia (Mic), macrophages (Macs), oligodendrocytes (Olgs), oligodendrocyte intermediate progenitor cells (IPCs), oligodendrocyte precursor cells (OPCs), endothelial cells (Ends), and fibroblasts (Fibs). To further validate the robustness of our clustering results, we additionally performed UMAP dimensionality reduction (Figure S1A), which revealed cell type segregation patterns highly consistent with those observed in the t-SNE analysis. Sample-wise t-SNE projections are provided in Figure S1B.

While neurons and mature oligodendrocytes remained the predominant populations in both groups, NTG treatment induced pronounced shifts in cellular composition (Figure 2E). Neurons declined from 40.2% in CON to 27.9% in NTG, whereas mature oligodendrocytes increased from 42.3% to 55.1%. Astrocytes, microglia, OPCs, and endothelial cells all showed modest decreases, while macrophages, fibroblasts, and IPCs expanded notably. These proportional changes were confirmed by the observed/expected ratios (Ro/e) (Figure 2F), suggesting that chronic NTG exposure perturbs glial homeostasis and promotes peripheral immune infiltration and extracellular matrix remodeling within the TNC.

To understand the functional consequences of these compositional shifts, we performed a DEG analysis across major cell types. In astrocytes, genes such as *Capn13* and *Lrrtm3* were upregulated, while multiple mitochondrial genes were downregulated. Microglia exhibited increased expression of *Wbp1l*, *Arhgap5*, and *Cdh23*, with reduced *Ctss*. Macrophages showed elevated *S100a8/a9*, *Napsa*, and *Hmgb2*, accompanied by the downregulation of *Mrc1*, *C1qa*, and *Apoe* (Figure 2G). Inflammation scoring using AUCell further revealed that the microglia, macrophages, and astrocytes displayed the highest inflammatory activity, whereas neurons remained largely quiescent (Figure 2H). These findings underscore glial and immune cell activation as central features of NTG-induced neuroinflammation.

Although neurons and oligodendrocytes exhibited compositional changes following NTG administration, our analysis deliberately focused on the glial contributions to CM pathophysiology, given their well-established roles in neuroinflammatory and neuroimmune signaling. Accordingly, we conducted detailed subclustering and trajectory analyses of the microglia and astrocytes to resolve their transcriptional heterogeneity and uncover functionally distinct reactive states.

### 3.3. NTG-Mic: A Proinflammatory Microglial Subpopulation Enriched in Glycolysis and Complement Activation

**Figure 3:**
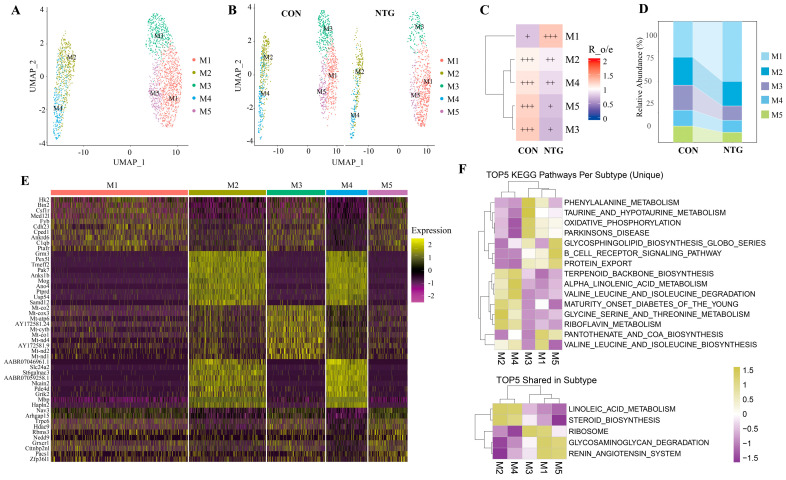
SnRNA-seq reveals NTG-induced microglia heterogeneity and distinct functions of each subtype. (**A**) UMAP visualization of 5 distinct microglia subtypes (M1–M5) pooled across groups, with each point representing an individual nucleus colored by subcluster identity. (**B**) Condition-specific microglia distribution shown in separate UMAP plots for CON (left) and NTG (right) groups. (**C**) Heatmap of subtype proportion alterations quantified by Ro/e ratio analysis. +++, Ro/e > 1; ++, 0.8 < Ro/e ≤ 1; +, 0.2 ≤ Ro/e ≤ 0.8. (**D**) Compositional changes of microglia subtypes between conditions. Stacked bars represent normalized cell proportions (total = 1). (**E**) Heatmap of top 10 DEGs across microglia subtypes. Rows represent genes; columns represent clusters. Color scale shows z-scored expression (dark yellow: upregulated; dark purple: downregulated). (**F**) Top 5 enriched KEGG pathways identified through GSVA analysis, showing a subtype-specific or shared functional signature.

To resolve the heterogeneity of microglial responses in CM, we re-clustered all the microglial nuclei from both NTG and CON groups, identifying five transcriptionally distinct subpopulations, designated M1 through M5 (Figure 3A,B). The relative abundance across these subpopulations differed markedly between groups: M1 underwent a notable expansion in NTG-treated rats, while M2 through M5 were relatively reduced (Figure 3C). The Ro/e ratios confirmed the selective enrichment of M1 in the NTG group (Figure 3D), leading us to designate M1 as NTG-associated Mic (NTG-Mic).

A differential expression analysis of the top ten markers per cluster revealed distinct molecular identities. NTG-Mic (M1) was characterized by the upregulation of glycolytic enzymes (e.g., *Hk2*), complement factors (e.g., *C1qb*), and inflammatory regulators (e.g., *Ptafr*, *Csf1r*). Among the other subclusters, M3 was enriched in mitochondrial genes (*Mt-Co1*, *Mt-Atp6*), andM5 exhibited glycosylation and immune-related signaling (e.g., *Zfp36l1*, *Trpc6*) (Figure 3E). Interestingly, M2 and M4 co-expressed oligodendrocyte markers such as *Mbp* and *Mog*, which may reflect (1) the uptake of myelin-derived transcripts via the phagocytosis of oligodendrocyte debris, as reported in demyelinating or neuroinflammatory contexts [30,31], or (2) transcriptomic dysregulation associated with dystrophic or disease-associated microglia in neurodegeneration [32,33].

To interrogate pathway-level functional differences, we performed GSVA across the five microglial subtypes (Figure 3F). M1 and M3 engaged biosynthesis and oxidative phosphorylation pathways, indicative of activated metabolic states. M2 and M4 emphasized lipid metabolism pathways (e.g., Steroid biosynthesis, Terpenoid backbone biosynthesis), suggesting roles in membrane remodeling. M5 was enriched for glycosylation and immune-related signaling (e.g., Glycosphingolipid biosynthesism B-cell receptor signaling), underscoring its potential immunomodulatory role.

These results reveal a spectrum of metabolic and immunologic phenotypes among microglial subtypes. The NTG-Mic (M1) population, in particular, exhibited strong activation signatures marked by the upregulation of glycolytic enzymes, complement components, and cytokine regulators. Comparative KEGG and GO enrichment analyses between NTG-Mic and the remaining microglia (M2-M5) further emphasized subtype-specific inflammatory and metabolic divergence (Figure S2).

### 3.4. Pseudotime Analysis Reveals Microglial Divergence Toward Inflammatory Fates

**Figure 4:**
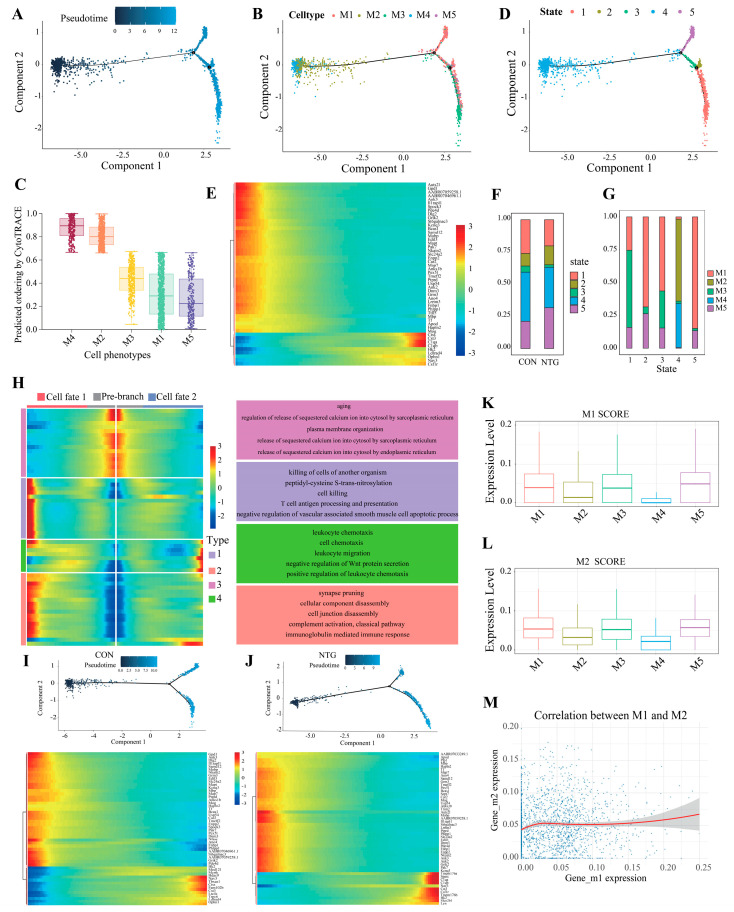
Pseudotemporal dynamics of NTG-induced microglial activation and fate determination. (**A**) Trajectory reconstruction revealing bifurcating differentiation paths. (**B**) Distribution of microglial subtypes (M1–M5) projected along the trajectory. (**C**) Differentiation potential inferred by CytoTRACE, with highest scores in M4 (root) cells. (**D**) Cell state assignments across pseudotime. (**E**) Heatmap of dynamic gene expression along the trajectory. (**F**) Comparison of pseudotime-defined cell state proportions between CON and NTG groups. (**G**) Relative abundance of microglial subtypes within each pseudotime-defined state. (**H**) Heatmap of branch-specific fate biases and their enriched functional pathways. (**I**,**J**) Condition-specific trajectories for CON and NTG groups, with corresponding gene expression patterns shown in heatmaps. (**K**,**L**) M1- and M2-like signature scores among microglial subtypes. (**M**) No correlation observed between M1 and M2 signature scores, indicating independent polarization trajectories.

To track the dynamic progression of microglial states under NTG challenge, we performed pseudotime trajectory analysis. Cells from the M4 subpopulation occupied the root of the trajectory, consistent with their highest progenitor-like score by CytoTRACE analysis, followed closely by M2, before bifurcating into two terminal branches: one leading to the metabolically specialized M3 subset and the other to a combined M1/M5 endpoint (Figure 4A–C). Notably, M1 cells were distributed across both branches, reflecting their transcriptional plasticity.

Mapping transcriptional states along the trajectory defined five discrete pseudotime-defined states (States 1–5) (Figure 4D). A heatmap of the top 50 trajectory-associated genes revealed stepwise transitions marked by complement activation (*C1qa*, *C1qb*), cytoskeletal remodeling (*Nav3*), and cytokine signaling (*Csf1r, Cst3*) (Figure 4E). Among these, state 5 was predominantly composed of M1 cells, whose abundance increased substantially following NTG treatment (Figure 4F,G).

Focusing on the second bifurcation point, we grouped branch-associated genes into two cell fates characterized by distinct gene modules (Figure 4H). The pre-branch state (state 4) expressed genes linked to cellular maintenance and calcium homeostasis, suggesting a preparatory phase prior to fate commitment. Fate 1 (lower branch, states 1 and 3) was defined by genes involved in immune activation and cell death processes, including antigen processing and immunoglobulin-mediated signaling. In contrast, fate 2 (upper branch, state 5) showed enrichment for genes associated with chemotaxis and immune cell migration, implicating this branch in inflammatory recruitment. A comparative analysis of the pseudotime trajectories in the CON (Figure 4I) and NTG (Figure 4J) groups revealed stimulus-dependent reprogramming. While key regulators such as *Hk2*, *Nav3*, and *Cst3* were shared across both conditions, the NTG-treated microglia exhibited gradual upregulation of *Tmem176a/b*, *Sparc*, *C1qa*, *C1qb*, and *Csf1r* along pseudotime, reinforcing the notion of NTG-driven inflammatory and metabolic activation.

Lastly, we applied M1/M2 polarization scoring across microglial clusters and found that M1, M3, and M5 co-expressed markers from both classical axes (Figure 4K,L), with no consistent inverse correlation between M1 and M2 scores (Figure 4M). This finding supports a growing consensus that traditional M1/M2 dichotomies are insufficient to capture the full complexity of microglial phenotypes, which are better represented as dynamic, trajectory-defined states.

### 3.5. NTG-Asts: A Reactive Astrocyte Subpopulation with Dual Inflammatory and Metabolic Programs

**Figure 5:**
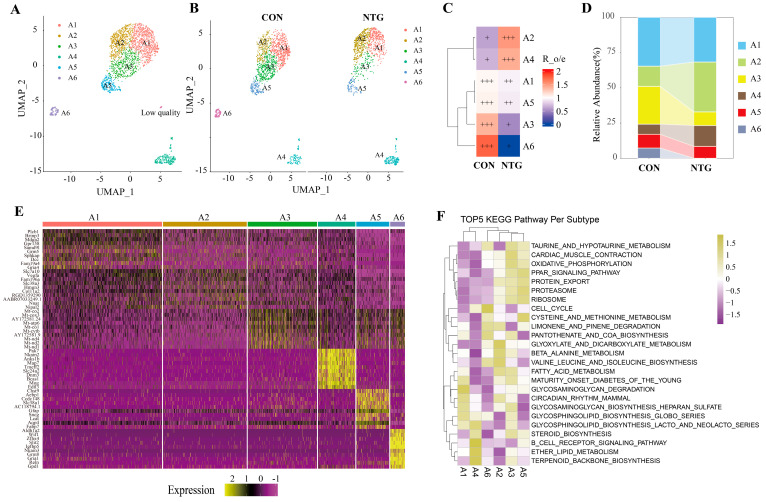
NTG-induced astrocytic heterogeneity and expansion of distinct reactive subtypes. (**A**) UMAP visualization of six transcriptionally distinct astrocytic subclusters (A1–A6). (**B**) Separate UMAP plots show astrocyte distribution in CON (left) and NTG (right) groups. (**C**) Ro/e ratios quantify the enrichment or depletion of each astrocyte subtype in NTG relative to CON. +++, Ro/e > 1; ++, 0.8 < Ro/e ≤ 1; +, 0.2 ≤ Ro/e ≤ 0.8. (**D**) Stacked bar plot displays the relative abundance of astrocytic subtypes between groups, highlighting the expansion of A2 and A4 under NTG. (**E**) Heatmap of top ranked genes delineates the molecular identity and specialization of each subtype. (**F**) GSVA-based KEGG pathway enrichment showing the top five functional pathways associated with each astrocytic subtype.

Following the approach used for microglia, we performed subclustering on astrocyte nuclei from the TNC, identifying six transcriptionally distinct subsets (A1–A6) (Figure 5A,B). NTG exposure significantly altered the composition of these subpopulations: A2 displayed the most robust expansion, followed by a moderate increase in A4, while A3 and A6 were markedly reduced (Figure 5C). The Ro/e ratios further confirmed the NTG-associated shifts in astrocyte composition (Figure 5D).

Integrated transcriptomic profiling and GSVA pathway analysis revealed that each subset harbored distinct molecular signatures corresponding to specialized functional roles (Figure 5E,F). A2, the most expanded population following NTG treatment, expressed *Slc7a10*, *Vegfa*, *Nnat*, and *Slc38a3* and was associated with pathways related to inflammation and amino acid metabolism, supporting its designation as the NTG-associated astrocyte (NTG-Ast). A4, marked by *Pak7*, *Tmeff2*, *Slc24a2*, and *Dnm3*, was enriched for T/B cell receptor signaling, NK cell-mediated cytotoxicity, and endocytosis, indicating immune and vesicular processing activity. A3, typified by mitochondrial genes (*Mt-co2*, *Mt-atp6*, *Mt-cytb*), was linked to oxidative phosphorylation and mitochondrial metabolism. A5, expressing canonical astrocyte markers *Gfap* and *Aqp4*, was enriched for glycosaminoglycan biosynthesis and gap junction signaling, consistent with a structural and homeostatic function. A6, defined by *Slit2* and *Aldh1a2*, showed enrichment in ECM-receptor interactions and Wnt signaling, suggestive of functions in tissue morphogenesis and glial remodeling.

Comparative KEGG and GO enrichment analyses between NTG-Ast subtypes (A2 and A4) and the remaining clusters further illustrated functional divergence, underscoring subtype-specific astrocytic adaptations to chronic NTG exposure (Figure S3).

### 3.6. Pseudotime Analysis Reveals Astrocytic Trajectories Toward Distinct Reactive States

**Figure 6:**
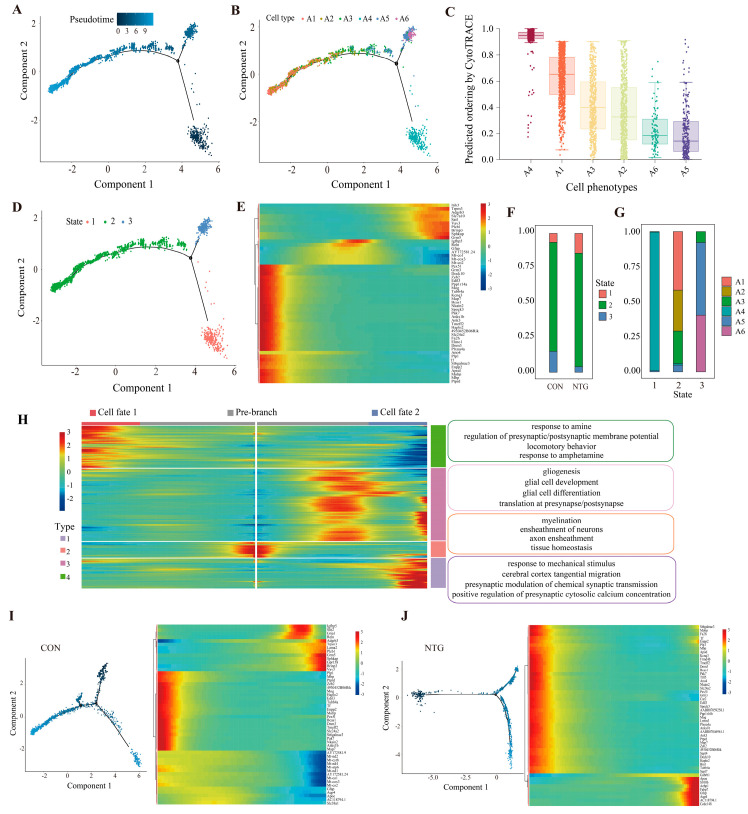
Pseudotemporal dynamics of NTG-induced astrocytic differentiation and fate specification. (**A**) Trajectory reconstruction shows bifurcating differentiation paths. (**B**) Distribution of astrocyte subtypes along the trajectory. (**C**) CytoTRACE scores indicate high progenitor-like potential in A4. (**D**) Cell state assignments derived from pseudotime. (**E**) Heatmap of the top 50 genes associated with pseudotime progression, showing distinct transcriptional shifts. (**F**,**G**) Distribution of astrocyte subtypes across pseudotime-defined states. (**H**) Branch-dependent gene modules categorized into four functional programs. (**I**,**J**) Comparative trajectory plots of CON and NTG astrocytes, with corresponding gene expression patterns.

Pseudotime trajectory analysis was performed to investigate the temporal dynamics of astrocyte activation in response to NTG. A4, which displayed a moderate NTG-induced expansion, was positioned at the root of the trajectory, consistent with its high progenitor-like potential as scored by CytoTRACE. From this origin, the trajectory bifurcated into two distinct branches: one leading to the NTG-associated A2 subset and the other toward terminally differentiated A5 and A6 states (Figure 6A–C). The concurrent expansion of A4 suggests that NTG not only promotes reactive subtypes but also recruits progenitor-like astrocytes as a reservoir for further differentiation.

Mapping the transcriptional progression revealed three major pseudotime states broadly corresponding to astrocyte subsets (Figure 6D). Heatmap visualization of the top 50 pseudotime-associated genes revealed progressive transitions marked by metabolic, structural, and synaptic regulation signatures (Figure 6E). A state distribution analysis showed that the A2 cells were exclusively aligned with state 2, while A6 corresponded solely to state 3, indicating distinct fate commitments (Figure 6F,G).

Branch-specific gene modules were grouped into four functional programs (Figure 6H): Module 1 (fate 1/state 2) included genes related to amine-mediated signaling, synaptic membrane potential regulation, and locomotor behavior, suggestive of a neuromodulatory phenotype; Module 2 (transition from root to fate 2) was enriched for gliogenesis and local protein synthesis, indicating an intermediate state supporting astrocytic differentiation; Module 3 (root/state 1) was associated with axon ensheathment and tissue maintenance, reflecting a progenitor-like homeostatic profile. Astrocytes have been shown to wrap unmyelinated axons and to express myelin-associated transcripts during early development [34,35], supporting this GO-based analysis; Module 4 (fate 2/state 3) was enriched for calcium signaling and synaptic transmission, indicating involvement in circuit plasticity and communication.

A comparative pseudotime analysis revealed that both NTG and CON astrocytes followed bifurcating trajectories; however, CON astrocytes exhibited greater topological complexity, including a subtle extended arc absent in the NTG group (Figure 6I,J). Despite the overall trajectory simplification, NTG astrocytes showed a sustained upregulation of reactive and gliotic markers, including *Apoe*, *S100b*, *Aebp1*, *Fabp7*, *Gfap*, and *Aqp4*, with *Apoe*, *Gfap*, and *Aqp4* exhibiting NTG-specific increases that diverged sharply from the control trends.

### 3.7. NTG Rewires Glial Communication Networks in the TNC

**Figure 7:**
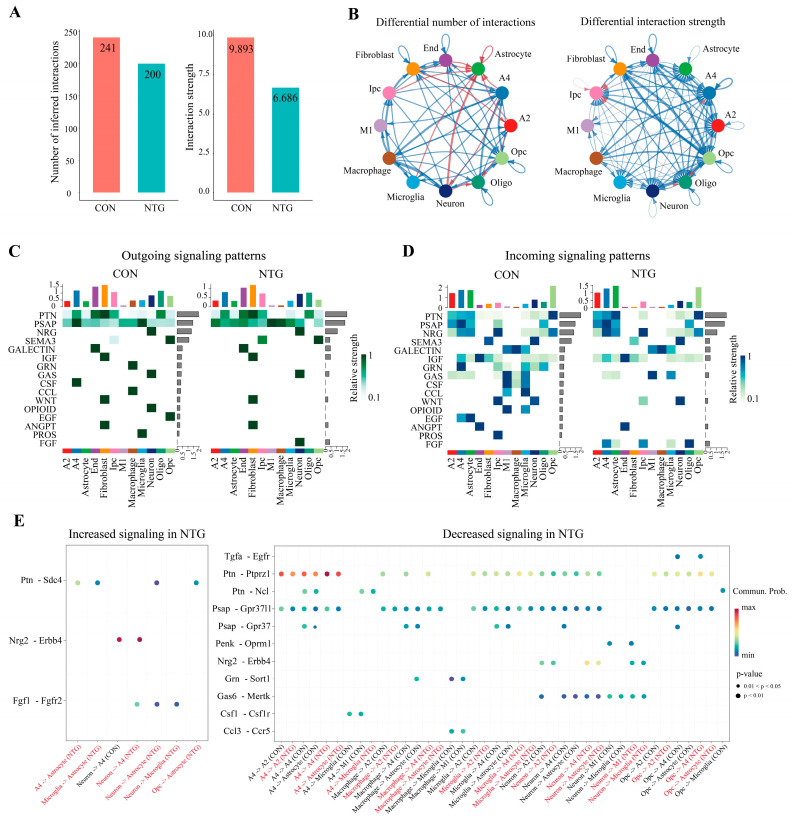
NTG rewires glial communication networks in the TNC. (**A**) Global reduction in the number and strength of cell–cell interactions in the NTG compared with CON. (**B**) CellChat analysis-based network visualization shows communication patterns across all cell types. (**C**) Heatmaps illustrating differences in the strength of outgoing and (**D**) incoming ligand–receptor-mediated interactions between cell types. (**E**) Dot plot highlights specific ligand–receptor pairs altered by NTG. Dot size reflects the *p*-value, and color indicates communication probability.

To examine how chronic NTG exposure alters intercellular signaling in the TNC, we applied CellChat analysis to infer ligand–receptor interactions across cell populations. Globally, the NTG-treated samples exhibited a marked reduction in both the number and overall strength of predicted interactions compared with controls (total interactions: 200 vs. 241; total strength: 6.686 vs. 9.893) (Figure 7A). This suggests a general suppression of intercellular communication under chronic migraine conditions, contributing to a functionally silenced and maladaptive neural microenvironment.

Despite this overall reduction, selected glial and neuronal subsets, particularly A2 and A4 astrocytes, microglia, and macrophages, showed increased interaction numbers, albeit with a weaker signaling strength, relative to other populations such as OPCs, fibroblasts, and endothelial cells (Figure 7B). This imbalance suggests compensatory or dysregulated glia-centric communication within the inflammatory niche.

The dissection of outgoing and incoming signaling profiles revealed that several canonical communication families, including GRN, CSF, CCL, and EGF, were broadly diminished in the NTG group (Figure 7C,D). Notably, only FGF signaling showed consistent upregulation across multiple cell types as incoming signals, particularly toward astrocytes. The ligand–receptor dot plot analysis further pinpointed individual pathways significantly altered by NTG (Figure 7E). Specifically, PSAP-GPR37/GPR37l1, CCL3-CCR5, GAS6-MERTK, GRN-SORT1, and CSF1-CSF1R interactions, predominantly from neurons, microglia, macrophages, and OPCs targeting A2 and A4 astrocytes and microglia, were strongly downregulated. In contrast, only a limited number of pathways, including FGF1-FGFR2, NRG2-ERBB4, and PTN-SDC4, were upregulated under NTG conditions, mainly originating from neurons and microglia and targeting astrocytes.

Together, chronic NTG exposure selectively rewires glial communication networks, amplifying inflammatory loops while suppressing homeostatic circuits, potentially contributing to sustained pain hypersensitivity and therapeutic resistance.

## 4. Discussion

This study presents the first single-nucleus transcriptomic atlas of the TNC in a CM rat model, focusing on glial responses. By integrating high-resolution cellular profiling, pseudotime trajectory mapping, and intercellular signaling analysis, we identified key NTG-responsive microglial and astrocytic subpopulations, delineated their activation pathways, and characterized their roles in reshaping neuroimmune intercellular communication. Together, these findings demonstrate that CM involves three interrelated glial processes: (1) the emergence of reactive subtypes; (2) skewed activation trajectories; and (3) rewired intercellular signaling, all contributing to sustained neuroinflammation. These insights deepen our understanding of CM pathophysiology by highlighting how glial heterogeneity and crosstalk fuel a persistent proinflammatory state within the central pain-processing network.

Chronic NTG exposure induced significant reorganization of the TNC glial landscape, marked by the expansion of distinct reactive microglial and astrocytic subpopulations. The NTG-responsive microglia (NTG-Mic) exhibited metabolic switching to glycolysis along with complement system activation, mirroring phenotypes characteristic of microglial activation in other chronic pain states [36,37]. In parallel, reactive astrocytes (NTG-Asts) upregulated both cytokine signaling pathways and amino acid transporters, suggesting dual roles in neuroinflammation and neuronal support [10,38]. Importantly, neither glial population conformed to traditional binary classification schemes—M1/M2 (microglia) and A1/A2 (astrocytes), instead developing context-specific hybrid states that reflect the unique pathophysiology of CM [39,40,41]. This spectrum of glial activation aligns with emerging models in neuroinflammatory and neurodegenerative disorders that underscore glial plasticity and state transitions, as revealed by single-cell analyses [42,43,44].

The functional implications of these activation states are profound. NTG-Ast upregulation of vascular endothelial growth factor (*Vegfa*) likely contributes to blood–brain barrier (BBB) disruption and subsequent immune cell infiltration, processes previously documented in migraine patients and rodent models [45,46]. Simultaneously, microglial complement activation (e.g., *C1qb*) and metabolic reprogramming (e.g., *Hk2*) may drive excessive synaptic pruning and neuronal sensitization [47,48]. Notably, the homeostatic astrocyte sublineage A6, which was enriched in extracellular matrix and Wnt signaling components, was virtually absent in NTG-treated animals. This loss of reparative glial subsets further undermines tissue resilience and restoration. Additionally, we observed an influx of peripheral macrophages into the NTG-treated TNC, indicating that both resident and recruited immune cells contribute to the amplification of neuroinflammation under compromised BBB conditions. Together, these changes establish a self-reinforcing cycle of glial dysfunction that perpetuates central sensitization.

The pseudotime trajectory analysis provided critical insights into the dynamic reprogramming of glial populations under CM conditions. NTG-Mic exhibited bifurcating differentiation paths, with one branch progressing toward a high-glycolytic, complement-enriched state and another retaining an oxidative phosphorylation-enriched profile. Similarly, astrocytes diverged toward either a reactive NTG-Ast phenotype or a more mature ECM-producing lineage observed in controls. This biased differentiation indicates that the CM microenvironment selectively expands proinflammatory glial subsets at the expense of protective ones. Similar trends of reparative glial attrition have been reported in neurodegenerative diseases, suggesting shared pathomechanisms across chronic CNS conditions [49,50].

Despite their ontogenetic differences, specific subpopulations of microglia (M3) and astrocytes (M3) demonstrated convergent transcriptional programs involving synaptic modulation and energy metabolism (e.g., *Gria4*, *Mog*, *Mt-co2*, *Mt-nd1*). This overlap may represent an initial compensatory response to neuronal stress that becomes maladaptive under chronic activation [51]. Such a shift from a protective to a pathogenic function likely plays a central role in sustaining hypersensitivity in migraine.

A major consequence of glial remodeling in CM is the reorganization of cell–cell communication networks. NTG exposure broadly suppressed neurotrophic and homeostatic signaling within the TNC microenvironment. Key interactions such as CSF1-CSF1R and GRN-SORT1 signaling, which help maintain microglial surveillance and restrain inflammation [50,52,53], were significantly diminished. Interestingly, although CSF1R expression was upregulated in NTG-Mic, astrocyte-to-microglia CSF1-CSF1R signaling was reduced, likely reflecting diminished ligand availability from astrocytes [54,55]. This disconnect suggests that microglia, while sensitized to trophic signals, are deprived of astrocyte-derived cues required for maintaining their homeostatic phenotype, potentially contributing to their shift toward a reactive, inflammatory state. Likewise, the reduction in CCL-CCR5 signaling may reflect impaired glial coordination and repair responses [56,57]. In contrast, proinflammatory and glia-centric pathways were selectively upregulated. FGF1-FGFR2 and PTN-SDC4 signaling, originating from activated microglia and targeting astrocytes, emerged as dominant communication routes. These pathways are known to promote astrocyte proliferation, inflammation, and ECM remodeling [58,59]. Their upregulation suggests the formation of a pathological microglia-to-astrocyte loop that sustains glial activation and potentially exacerbates BBB disruption. This rewiring transforms the TNC from a regulated neuroimmune interface into a hyperreactive glial niche characterized by unrestrained crosstalk and nociceptive amplification. These findings are consistent with evolving theories of migraine chronification, which emphasize the disinhibition of glial–neuronal circuits and the loss of anti-nociceptive “brakes” [60,61,62]. Our analysis not only identifies the key glial subtypes involved in this transition but also specifies actionable ligand–receptor pathways, offering mechanistic insights and novel targets for therapeutic intervention.

Several limitations of this study should be acknowledged. First, as inherent to all snRNA-seq approaches, our dataset captures primarily nuclear transcripts, potentially underrepresenting cytoplasmic or rapidly induced genes involved in acute signaling pathways. Second, the exclusive use of male rats to avoid hormonal confounders limits generalizability. Since migraine disproportionately affects females and sex-dependent glial transcriptomic responses have been reported, validation in female models is critical. Third, our analysis was restricted to a single chronic timepoint (day 9 post-NTG), which precludes insights into the dynamic evolution of glial states over the course of migraine progression or resolution. Longitudinal transcriptomic profiling is needed to track the emergence, plasticity, and potential reversibility of NTG-induced subtypes such as NTG-Mic and NTG-Asts. Fourth, the predicted intercellular interactions inferred from transcriptomic data are inherently limited by the absence of spatial resolution and protein-level confirmation. Because the nuclei of sender and receiver cell types are not co-localized within the original tissue architecture, these inferences remain correlative and cannot establish causality. Furthermore, mRNA expression levels do not always correlate with protein abundance or functional activity. Future studies integrating spatial transcriptomics, multiplexed immunohistochemistry, or single-cell proteomics will be essential to validate and refine these predicted ligand–receptor signaling networks. Lastly, this study focused exclusively on protein-coding transcripts. Non-coding RNAs, including microRNAs, and circular RNAs, were not captured, despite their known roles in regulating glial activation and neuroinflammatory cascades. Future multi-omics approaches that integrate transcriptomics with epigenomic and proteomic layers will provide a more comprehensive understanding of the glial regulatory architecture in chronic migraine.

Taken together, our findings uncover multiple mechanisms for therapeutic intervention. Strategies to restore homeostatic ligand–receptor interactions, such as GRN or CSF1 supplementation, may re-establish microglial quiescence. Conversely, blocking proinflammatory axes like FGF1-FGFR2 or PTN-SDC4 could interrupt the pathological microglia–astrocyte loop. Additionally, targeting metabolic reprogramming through glycolysis inhibition or mitochondrial support may mitigate NTG-induced glial hyperactivation. Given the observed plasticity in glial trajectories, the re-differentiation of reactive subtypes into protective phenotypes represents a promising therapeutic avenue.

## 5. Conclusions

In summary, this study uncovers a glia-centric dimension of CM pathophysiology, showing that NTG profoundly reprograms the microglial and astrocytic states, perturbs the metabolic–immune balance, and disrupts intercellular signaling in the TNC. These findings establish the glia as central orchestrators of migraine chronification and offer a conceptual framework for future mechanistic investigations and glia-targeted therapeutic strategies that go beyond traditional neuron- or vascular-focused paradigms.

## Data Availability

Due to laboratory safety regulations, some datasets involved in this study are not fully publicly available. Reasonable requests for relevant data can be directed to the corresponding author upon reasonable request.

## Supplementary Materials

The following supporting information can be downloaded at https://www.mdpi.com/article/10.3390/biom15070942/s1: Figure S1: UMAP and sample-wise t-SNE projections of annotated cell populations; Figure S2: Volcano plot and comparative enrichment analysis of NTG-Mic versus other microglial clusters; Figure S3: Volcano plot and comparative enrichment analysis of NTG-Ast subtypes versus other astrocyte clusters..

## Abbreviations

The following abbreviations are used in this manuscript:

CM	Chronic migraine
CNS	Central nervous system
GO	Gene ontology
GSVA	Gene set variation analysis
KEGG	Kyoto encyclopedia of genes and genomes
NTG	Nitroglycerin
PPI	Protein–protein interaction
PWMT	Paw withdrawal mechanical threshold
TFT	Tail-flick test
TNC	Trigeminal nucleus caudalis
snRNA	Single-nucleus RNA sequencing

## References

[1] May A., Schulte L.H. (2016). Chronic migraine: Risk factors, mechanisms and treatment. Nat. Rev. Neurol..

[2] Ashina M., Katsarava Z., Do T.P., Buse D.C., Pozo-Rosich P., Özge A., Krymchantowski A.V., Lebedeva E.R., Ravishankar K., Yu S. (2021). Migraine: Epidemiology and systems of care. Lancet.

[3] Yeh P.K., An Y.C., Hung K.S., Yang F.C. (2024). Influences of Genetic and Environmental Factors on Chronic Migraine: A Narrative Review. Curr. Pain Headache Rep..

[4] Ha W.S., Chu M.K. (2024). Altered immunity in migraine: A comprehensive scoping review. J. Headache Pain.

[5] Ashina M., Hansen J.M., Do T.P., Melo-Carrillo A., Burstein R., Moskowitz M.A. (2019). Migraine and the trigeminovascular system-40 years and counting. Lancet Neurol..

[6] Sacristan C. (2020). Microglia and Astrocyte Crosstalk in Immunity. Trends Immunol..

[7] Linnerbauer M., Wheeler M.A., Quintana F.J. (2020). Astrocyte Crosstalk in CNS Inflammation. Neuron.

[8] Stratoulias V., Venero J.L., Tremblay M., Joseph B. (2019). Microglial subtypes: Diversity within the microglial community. EMBO J..

[9] Sun S., Fan Z., Liu X., Wang L., Ge Z. (2024). Microglia TREM1-mediated neuroinflammation contributes to central sensitization via the NF-kappaB pathway in a chronic migraine model. J. Headache Pain.

[10] Zhao J., Blaeser A.S., Levy D. (2021). Astrocytes mediate migraine-related intracranial meningeal mechanical hypersensitivity. Pain.

[11] Wei S., Du T., Zhang L., Li X., Wang Z., Ning Y., Tang Y., Wu X., Han J. (2024). A comprehensive exploration of astrocytes in migraine: A bibliometric and visual analysis. Eur. J. Med. Res..

[12] Mathys H., Boix C.A., Akay L.A., Xia Z., Davila-Velderrain J., Ng A.P., Jiang X., Abdelhady G., Galani K., Mantero J. (2024). Single-cell multiregion dissection of Alzheimer’s disease. Nature.

[13] Renthal W. (2018). Localization of migraine susceptibility genes in human brain by single-cell RNA sequencing. Cephalalgia.

[14] Vgontzas A., Renthal W. (2019). Migraine-associated gene expression in cell types of the central and peripheral nervous system. Cephalalgia.

[15] Chen L., Li Y., Zhu L., Jin H., Kang X., Feng Z. (2023). Single-cell RNA sequencing in the context of neuropathic pain: Progress, challenges, and prospects. Transl. Res..

[16] Yang L., Xu M., Bhuiyan S.A., Li J., Zhao J., Cohrs R.J., Susterich J.T., Signorelli S., Green U., Stone J.R. (2022). Human and mouse trigeminal ganglia cell atlas implicates multiple cell types in migraine. Neuron.

[17] Sureda-Gibert P., Romero-Reyes M., Akerman S. (2022). Nitroglycerin as a model of migraine: Clinical and preclinical review. Neurobiol. Pain.

[18] Demartini C., Greco R., Zanaboni A.M., Sances G., De Icco R., Borsook D., Tassorelli C. (2019). Nitroglycerin as a comparative experimental model of migraine pain: From animal to human and back. Prog. Neurobiol..

[19] Zhang Y., Zhang Y., Tian K., Wang Y., Fan X., Pan Q., Qin G., Zhang D., Chen L., Zhou J. (2020). Calcitonin gene-related peptide facilitates sensitization of the vestibular nucleus in a rat model of chronic migraine. J. Headache Pain.

[20] Dobin A., Davis C.A., Schlesinger F., Drenkow J., Zaleski C., Jha S., Batut P., Chaisson M., Gingeras T.R. (2013). STAR: Ultrafast universal RNA-seq aligner. Bioinformatics.

[21] McGinnis C.S., Murrow L.M., Gartner Z.J. (2019). DoubletFinder: Doublet Detection in Single-Cell RNA Sequencing Data Using Artificial Nearest Neighbors. Cell Syst..

[22] Yang S., Corbett S.E., Koga Y., Wang Z., Johnson W.E., Yajima M., Campbell J.D. (2020). Decontamination of ambient RNA in single-cell RNA-seq with DecontX. Genome Biol..

[23] Hao Y., Hao S., Andersen-Nissen E., Mauck W.M., Zheng S., Butler A., Lee M.J., Wilk A.J., Darby C., Zager M. (2021). Integrated analysis of multimodal single-cell data. Cell.

[24] Hu C., Li T., Xu Y., Zhang X., Li F., Bai J., Chen J., Jiang W., Yang K., Ou Q. (2022). CellMarker 2.0: An updated database of manually curated cell markers in human/mouse and web tools based on scRNA-seq data. Nucleic Acids Res..

[25] Valihrach L., Matusova Z., Zucha D., Klassen R., Benesova S., Abaffy P., Kubista M., Anderova M. (2022). Recent advances in deciphering oligodendrocyte heterogeneity with single-cell transcriptomics. Front. Cell. Neurosci..

[26] Marques S., Zeisel A., Codeluppi S., van Bruggen D., Mendanha Falcão A., Xiao L., Li H., Häring M., Hochgerner H., Romanov R.A. (2016). Oligodendrocyte heterogeneity in the mouse juvenile and adult central nervous system. Science.

[27] Cheng S., Li Z., Gao R., Xing B., Gao Y., Yang Y., Qin S., Zhang L., Ouyang H., Du P. (2021). A pan-cancer single-cell transcriptional atlas of tumor infiltrating myeloid cells. Cell.

[28] Trapnell C., Cacchiarelli D., Grimsby J., Pokharel P., Li S., Morse M., Lennon N.J., Livak K.J., Mikkelsen T.S., Rinn J.L. (2014). The dynamics and regulators of cell fate decisions are revealed by pseudotemporal ordering of single cells. Nat. Biotechnol..

[29] Jin S., Guerrero-Juarez C.F., Zhang L., Chang I., Ramos R., Kuan C.H., Myung P., Plikus M.V., Nie Q. (2021). Inference and analysis of cell-cell communication using CellChat. Nat. Commun..

[30] Jordão M.J.C., Sankowski R., Brendecke S.M., Sagar, Locatelli G., Tai Y.H., Tay T.L., Schramm E., Armbruster S., Hagemeyer N. (2019). Single-cell profiling identifies myeloid cell subsets with distinct fates during neuroinflammation. Science.

[31] Safaiyan S., Besson-Girard S., Kaya T., Cantuti-Castelvetri L., Liu L., Ji H., Schifferer M., Gouna G., Usifo F., Kannaiyan N. (2021). White matter aging drives microglial diversity. Neuron.

[32] Gao C., Jiang J., Tan Y., Chen S. (2023). Microglia in neurodegenerative diseases: Mechanism and potential therapeutic targets. Signal Transduct. Target. Ther..

[33] Srinivasan K., Friedman B.A., Etxeberria A., Huntley M.A., van der Brug M.P., Foreman O., Paw J.S., Modrusan Z., Beach T.G., Serrano G.E. (2020). Alzheimer’s Patient Microglia Exhibit Enhanced Aging and Unique Transcriptional Activation. Cell Rep..

[34] Heller D.T., Kolson D.R., Brandebura A.N., Amick E.M., Wan J., Ramadan J., Holcomb P.S., Liu S., Deerinck T.J., Ellisman M.H. (2024). Astrocyte ensheathment of calyx-forming axons of the auditory brainstem precedes accelerated expression of myelin genes and myelination by oligodendrocytes. J. Comp. Neurol..

[35] Alghamdi B., Fern R. (2015). Phenotype overlap in glial cell populations: Astroglia, oligodendroglia and NG-2(+) cells. Front. Neuroanat..

[36] Cropper H.C., Conway C.M., Wyche W., Pradhan A.A. (2024). Glial activation in pain and emotional processing regions in the nitroglycerin mouse model of chronic migraine. Headache: J. Head Face Pain.

[37] He W., Long T., Pan Q., Zhang S., Zhang Y., Zhang D., Qin G., Chen L., Zhou J. (2019). Microglial NLRP3 inflammasome activation mediates IL-1β release and contributes to central sensitization in a recurrent nitroglycerin-induced migraine model. J. Neuroinflamm..

[38] Hasel P., Dando O., Jiwaji Z., Baxter P., Todd A.C., Heron S., Márkus N.M., McQueen J., Hampton D.W., Torvell M. (2017). Neurons and neuronal activity control gene expression in astrocytes to regulate their development and metabolism. Nat. Commun..

[39] Hu X., Leak R.K., Shi Y., Suenaga J., Gao Y., Zheng P., Chen J. (2015). Microglial and macrophage polarization—New prospects for brain repair. Nat. Rev. Neurol..

[40] Lawrence J.M., Schardien K., Wigdahl B., Nonnemacher M.R. (2023). Roles of neuropathology-associated reactive astrocytes: A systematic review. Acta Neuropathol. Commun..

[41] Shao F., Wang X., Wu H., Wu Q., Zhang J. (2022). Microglia and Neuroinflammation: Crucial Pathological Mechanisms in Traumatic Brain Injury-Induced Neurodegeneration. Front. Aging Neurosci..

[42] Batiuk M.Y., Martirosyan A., Wahis J., de Vin F., Marneffe C., Kusserow C., Koeppen J., Viana J.F., Oliveira J.F., Voet T. (2020). Identification of region-specific astrocyte subtypes at single cell resolution. Nat. Commun..

[43] Li D., Yang K., Li J., Xu X., Gong L., Yue S., Wei H., Yue Z., Wu Y., Yin S. (2024). Single-cell sequencing reveals glial cell involvement in development of neuropathic pain via myelin sheath lesion formation in the spinal cord. J. Neuroinflamm..

[44] Li H., Liu P., Zhang B., Yuan Z., Guo M., Zou X., Qian Y., Deng S., Zhu L., Cao X. (2023). Acute ischemia induces spatially and transcriptionally distinct microglial subclusters. Genome Med..

[45] Schain A.J., Melo-Carrillo A., Borsook D., Grutzendler J., Strassman A.M., Burstein R. (2018). Activation of pial and dural macrophages and dendritic cells by cortical spreading depression. Ann. Neurol..

[46] Khan S., Amin F.M., Fliedner F.P., Christensen C.E., Tolnai D., Younis S., Olinger A.C.R., Birgens H., Daldrup-Link H., Kjær A. (2019). Investigating macrophage-mediated inflammation in migraine using ultrasmall superparamagnetic iron oxide-enhanced 3T magnetic resonance imaging. Cephalalgia.

[47] Stevens B., Allen N.J., Vazquez L.E., Howell G.R., Christopherson K.S., Nouri N., Micheva K.D., Mehalow A.K., Huberman A.D., Stafford B. (2007). The Classical Complement Cascade Mediates CNS Synapse Elimination. Cell.

[48] Baik S.H., Kang S., Lee W., Choi H., Chung S., Kim J.I., Mook-Jung I. (2019). A Breakdown in Metabolic Reprogramming Causes Microglia Dysfunction in Alzheimer’s Disease. Cell Metab..

[49] Zamboni M., Llorens-Bobadilla E., Magnusson J.P., Frisén J. (2020). A Widespread Neurogenic Potential of Neocortical Astrocytes Is Induced by Injury. Cell Stem Cell.

[50] Matusova Z., Hol E.M., Pekny M., Kubista M., Valihrach L. (2023). Reactive astrogliosis in the era of single-cell transcriptomics. Front. Cell. Neurosci..

[51] Liddelow S.A., Guttenplan K.A., Clarke L.E., Bennett F.C., Bohlen C.J., Schirmer L., Bennett M.L., Münch A.E., Chung W.-S., Peterson T.C. (2017). Neurotoxic reactive astrocytes are induced by activated microglia. Nature.

[52] Xiang C., Li H., Tang W. (2023). Targeting CSF-1R represents an effective strategy in modulating inflammatory diseases. Pharmacol. Res..

[53] Jian J., Konopka J., Liu C. (2013). Insights into the role of progranulin in immunity, infection, and inflammation. J. Leukoc. Biol..

[54] Wang Y., Szretter K.J., Vermi W., Gilfillan S., Rossini C., Cella M., Barrow A.D., Diamond M.S., Colonna M. (2012). IL-34 is a tissue-restricted ligand of CSF1R required for the development of Langerhans cells and microglia. Nat. Immunol..

[55] Nandi S., Gokhan S., Dai X.-M., Wei S., Enikolopov G., Lin H., Mehler M.F., Stanley E.R. (2012). The CSF-1 receptor ligands IL-34 and CSF-1 exhibit distinct developmental brain expression patterns and regulate neural progenitor cell maintenance and maturation. Dev. Biol..

[56] Lin J., Xu Y., Guo P., Chen Y.J., Zhou J., Xia M., Tan B., Liu X., Feng H., Chen Y. (2023). CCL5/CCR5-mediated peripheral inflammation exacerbates blood–brain barrier disruption after intracerebral hemorrhage in mice. J. Transl. Med..

[57] Zeng Z., Lan T., Wei Y., Wei X. (2022). CCL5/CCR5 axis in human diseases and related treatments. Genes. Dis..

[58] Song Y., Li H., Li Y., Xu H., Nazir F.H., Jiang W., Zheng L., Tang C. (2025). Astrocyte-derived PTN alleviates deficits in hippocampal neurogenesis and cognition in models of multiple sclerosis. Stem Cell Rep..

[59] Xie Y., Su N., Yang J., Tan Q., Huang S., Jin M., Ni Z., Zhang B., Zhang D., Luo F. (2020). FGF/FGFR signaling in health and disease. Signal Transduct. Target. Ther..

[60] Vila-Pueyo M., Gliga O., Gallardo V.J., Pozo-Rosich P. (2023). The Role of Glial Cells in Different Phases of Migraine: Lessons from Preclinical Studies. Int. J. Mol. Sci..

[61] Noseda R., Burstein R. (2013). Migraine pathophysiology: Anatomy of the trigeminovascular pathway and associated neurological symptoms, CSD, sensitization and modulation of pain. Pain.

[62] Zhang J., Simoes R., Guo T., Cao Y.Q. (2024). Neuroimmune interactions in the development and chronification of migraine headache. Trends Neurosci..

