# Supplementary figures and images for: Single-Nucleus Transcriptomics Reveals Glial Metabolic–Immune Rewiring and Intercellular Signaling Disruption in Chronic Migraine
Source: Biomolecules. 2025 Jun 28;15(7):942. doi: 10.3390/biom15070942 (PMC12292200; doi:10.3390/biom15070942)

A

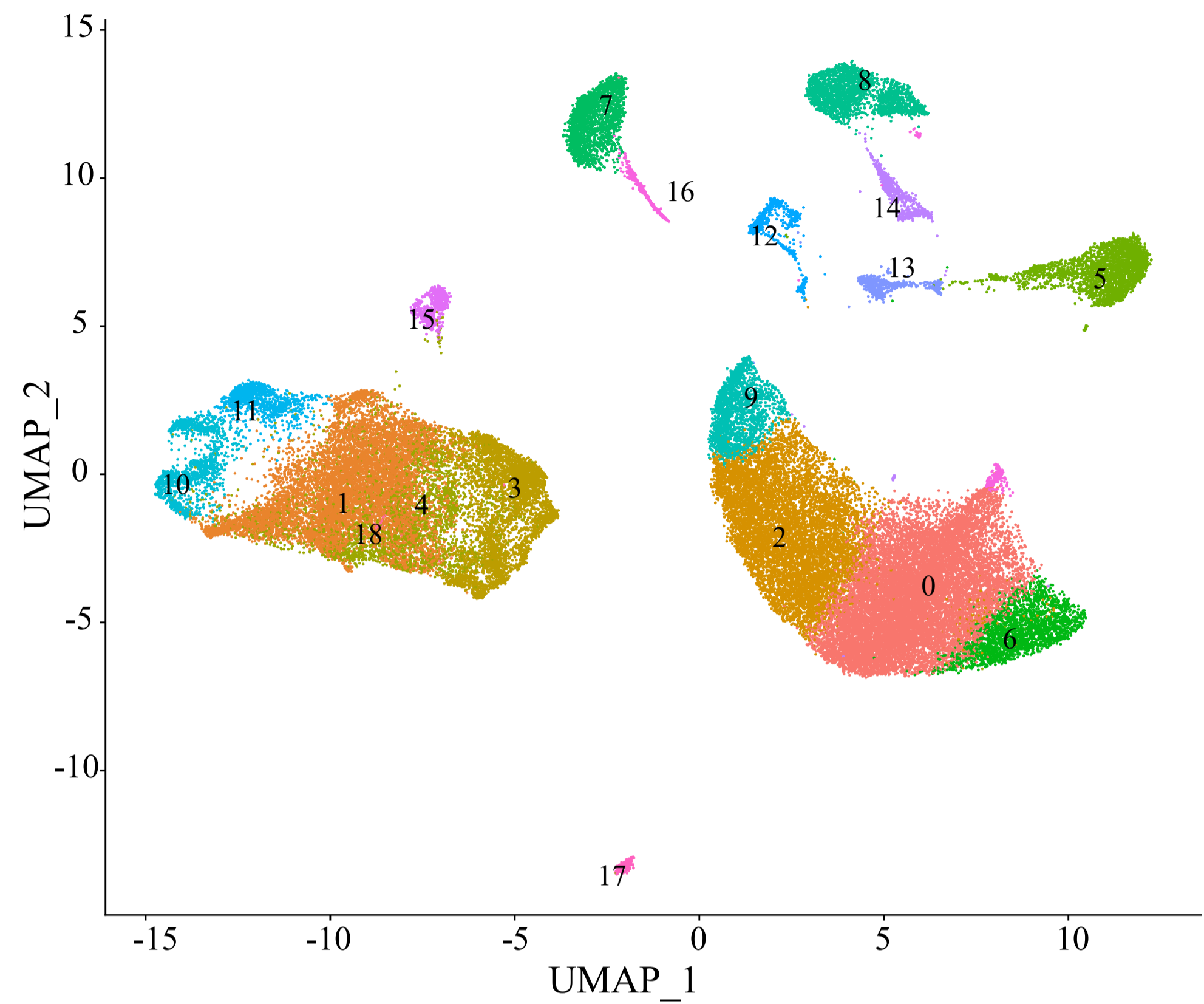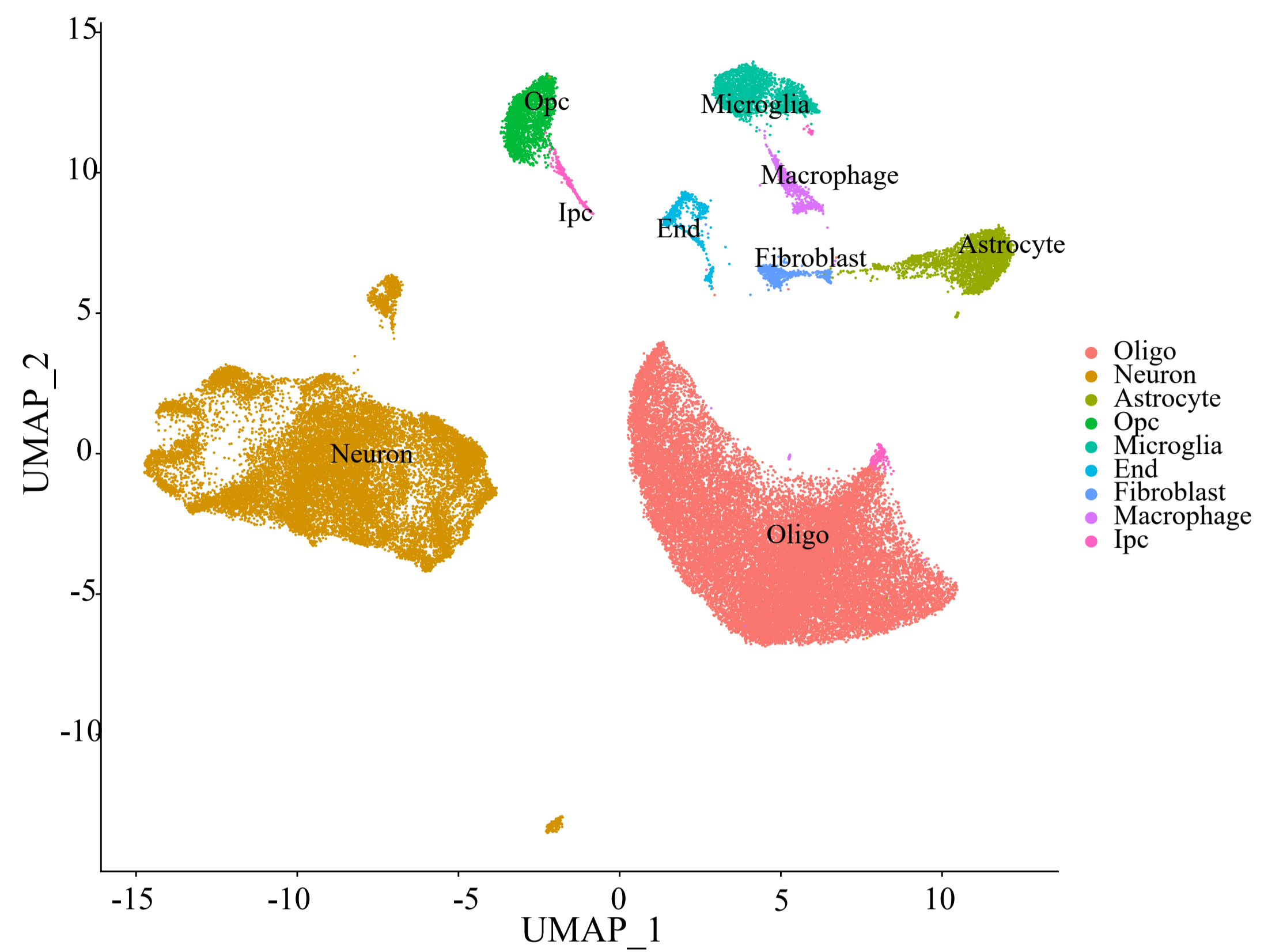

B

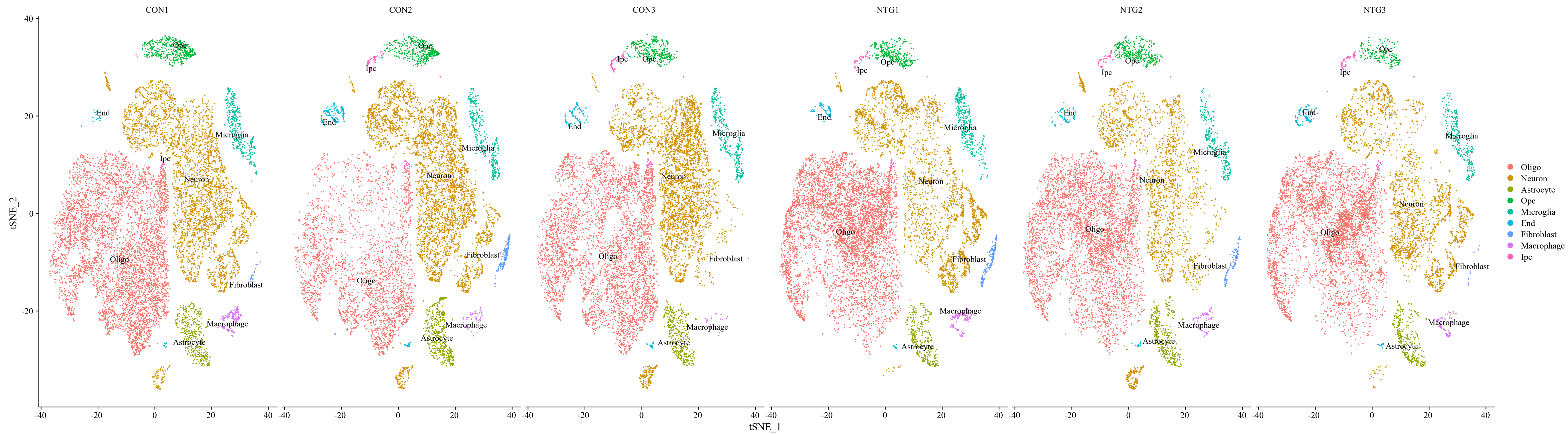

Supplement: Supplementary file 1 [file biomolecules-15-00942-s001.zip › supplementary Figure S1.pdf]

NTG-Mic vs Other-Mic

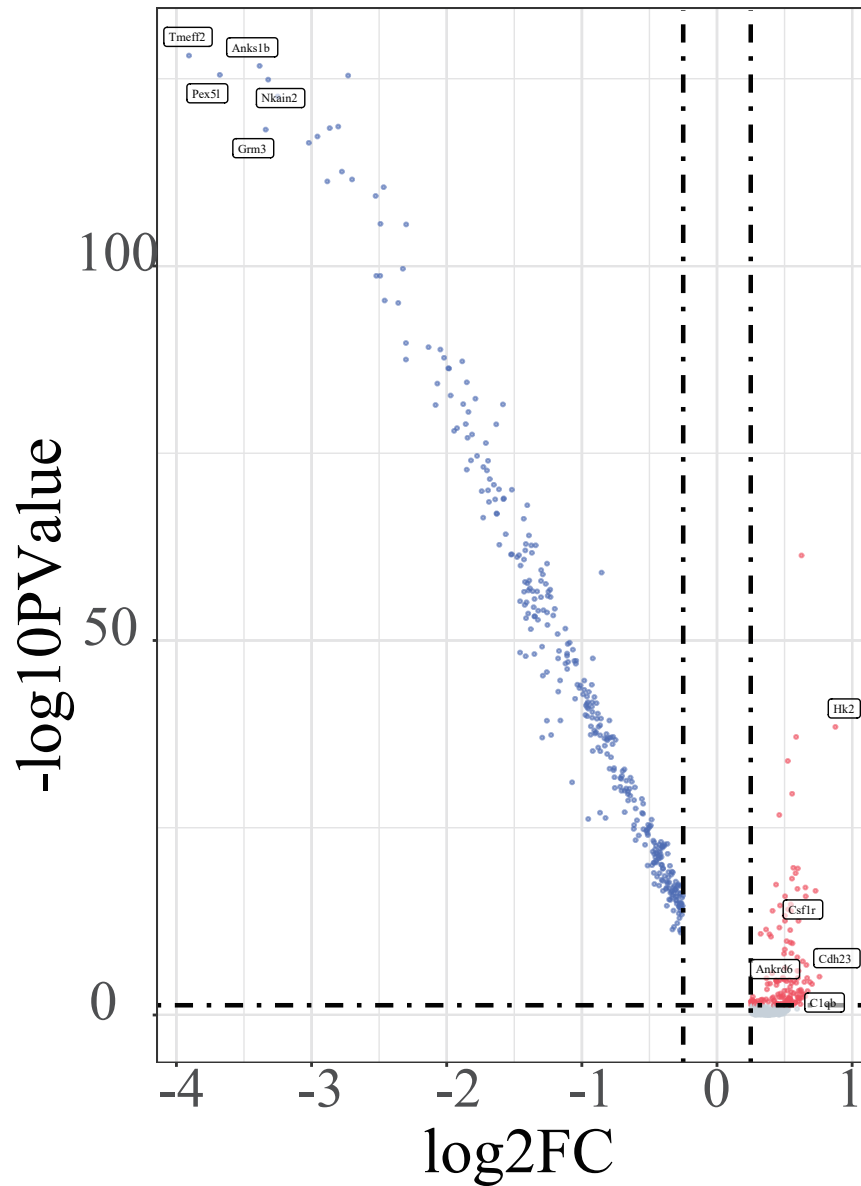

- Down
- Normal
- Up

Gene Ontology enrichment

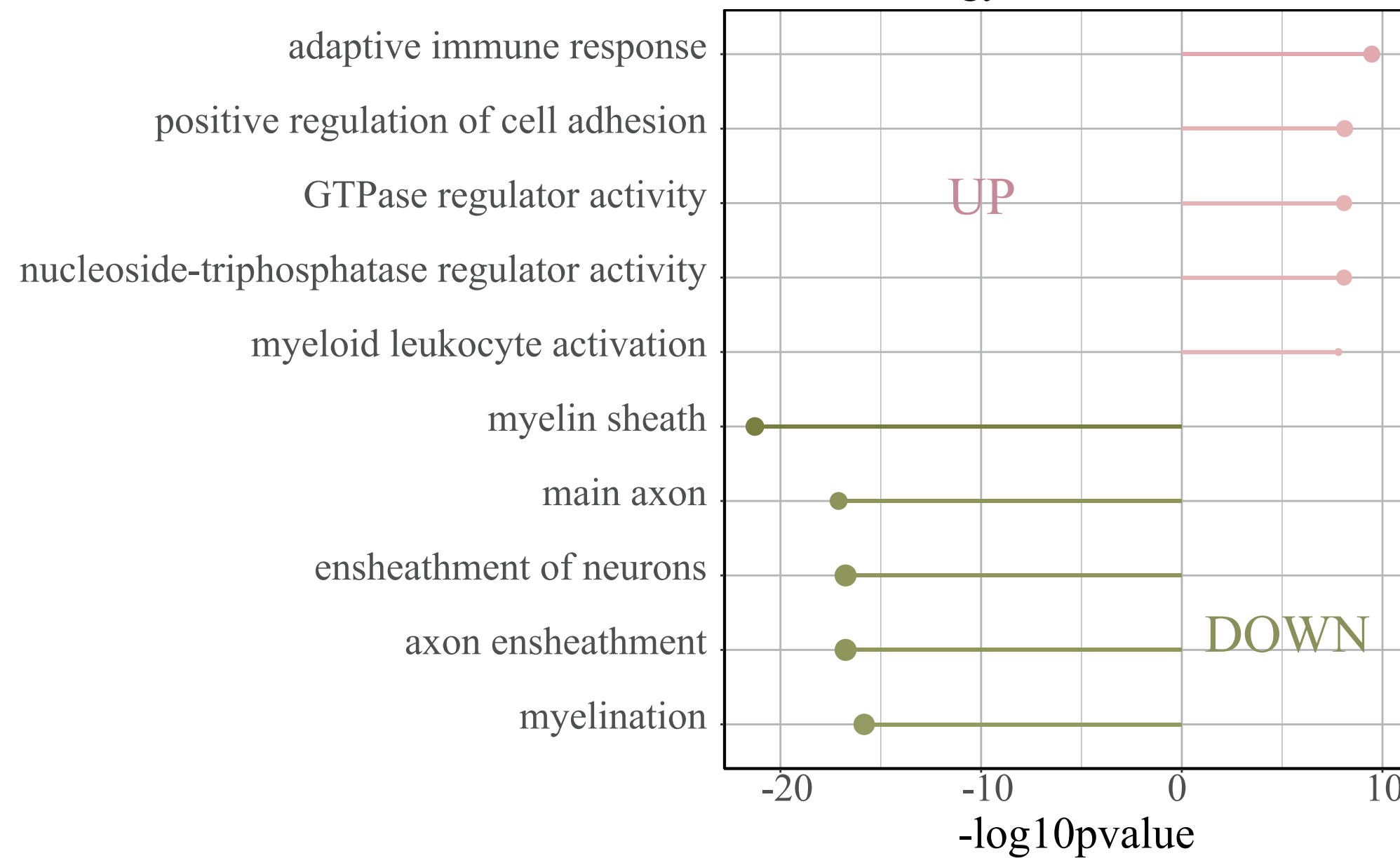

KEGG enrichment

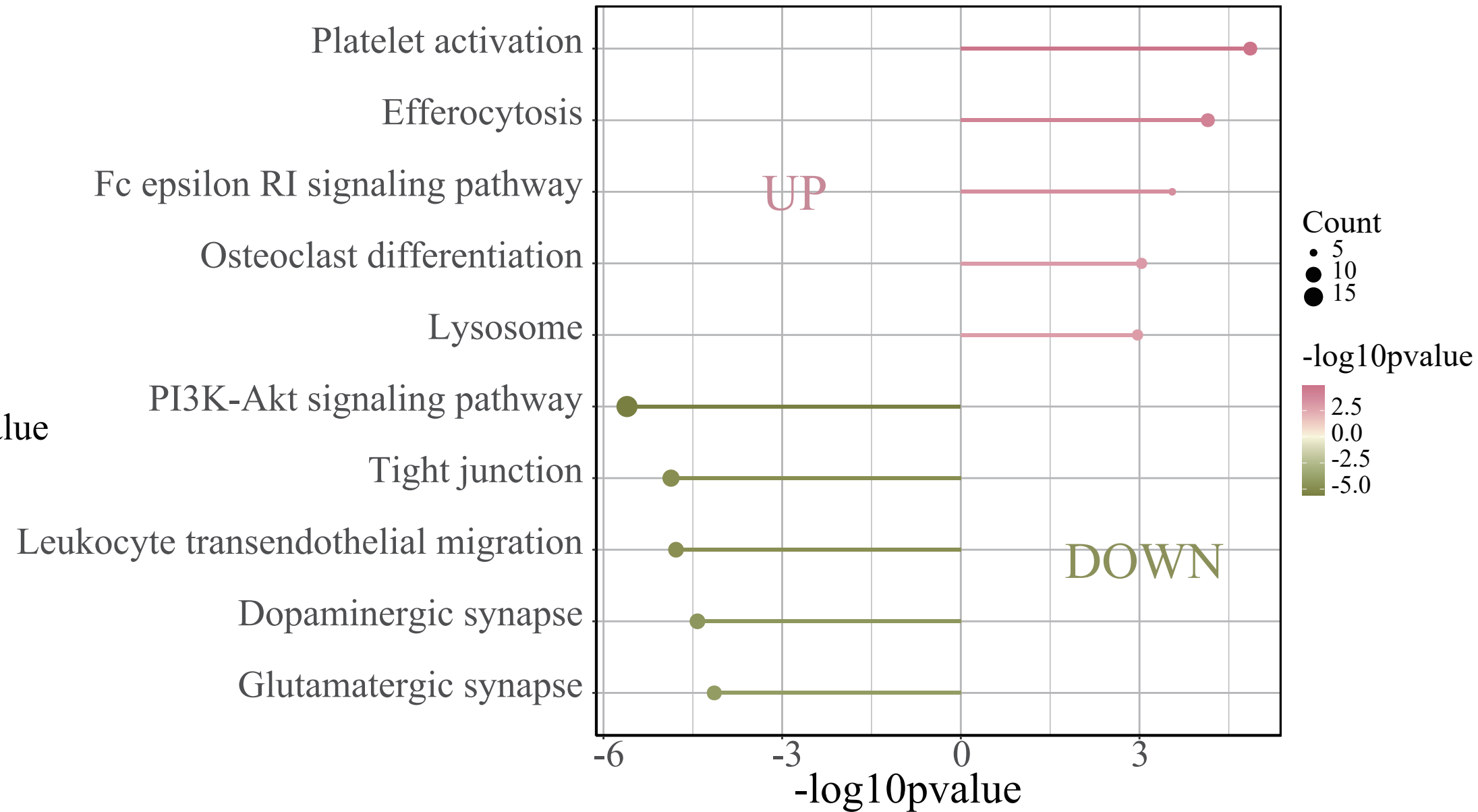

Supplement: Supplementary file 1 [file biomolecules-15-00942-s001.zip › Supplementary Figure S2.pdf]

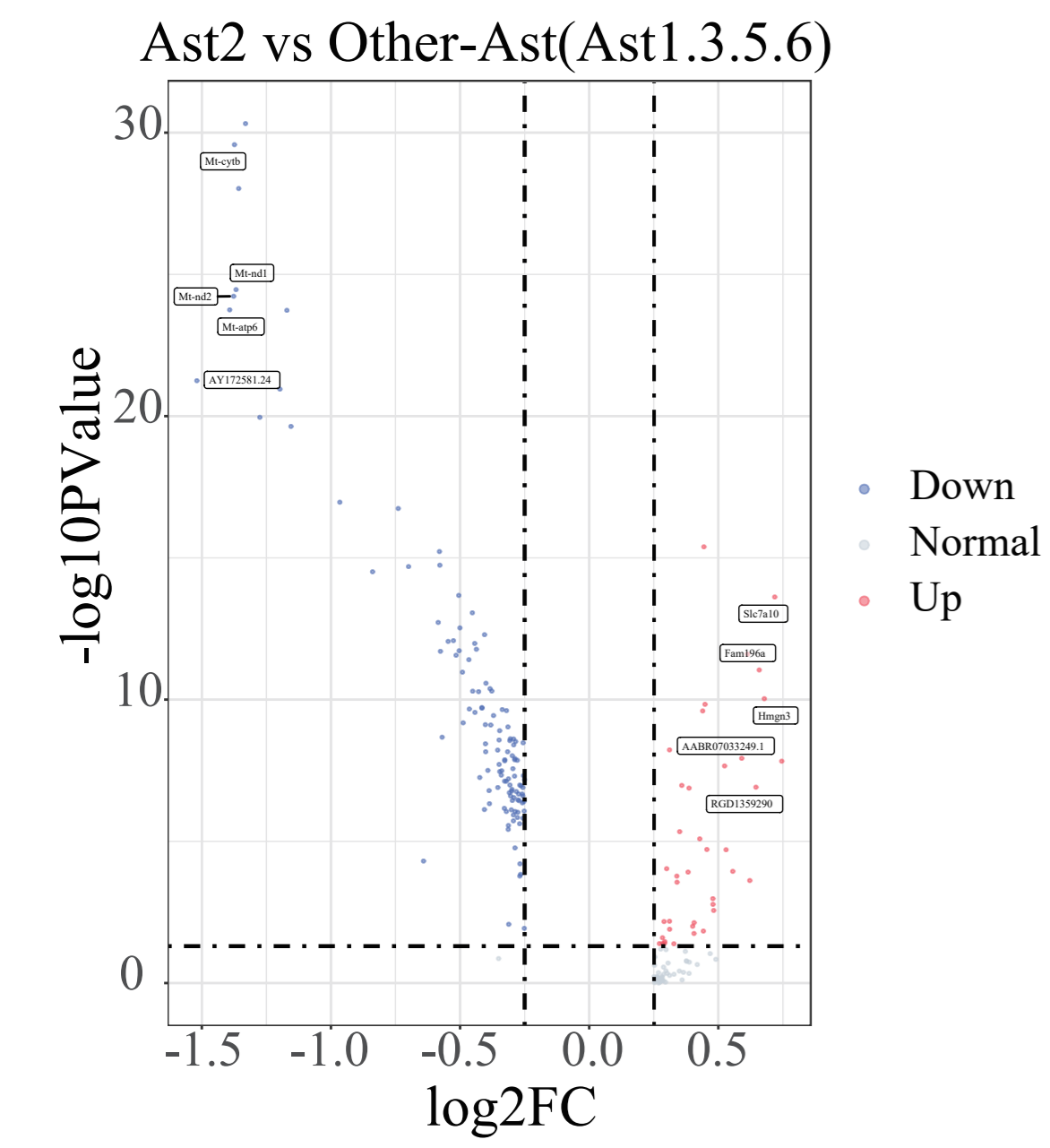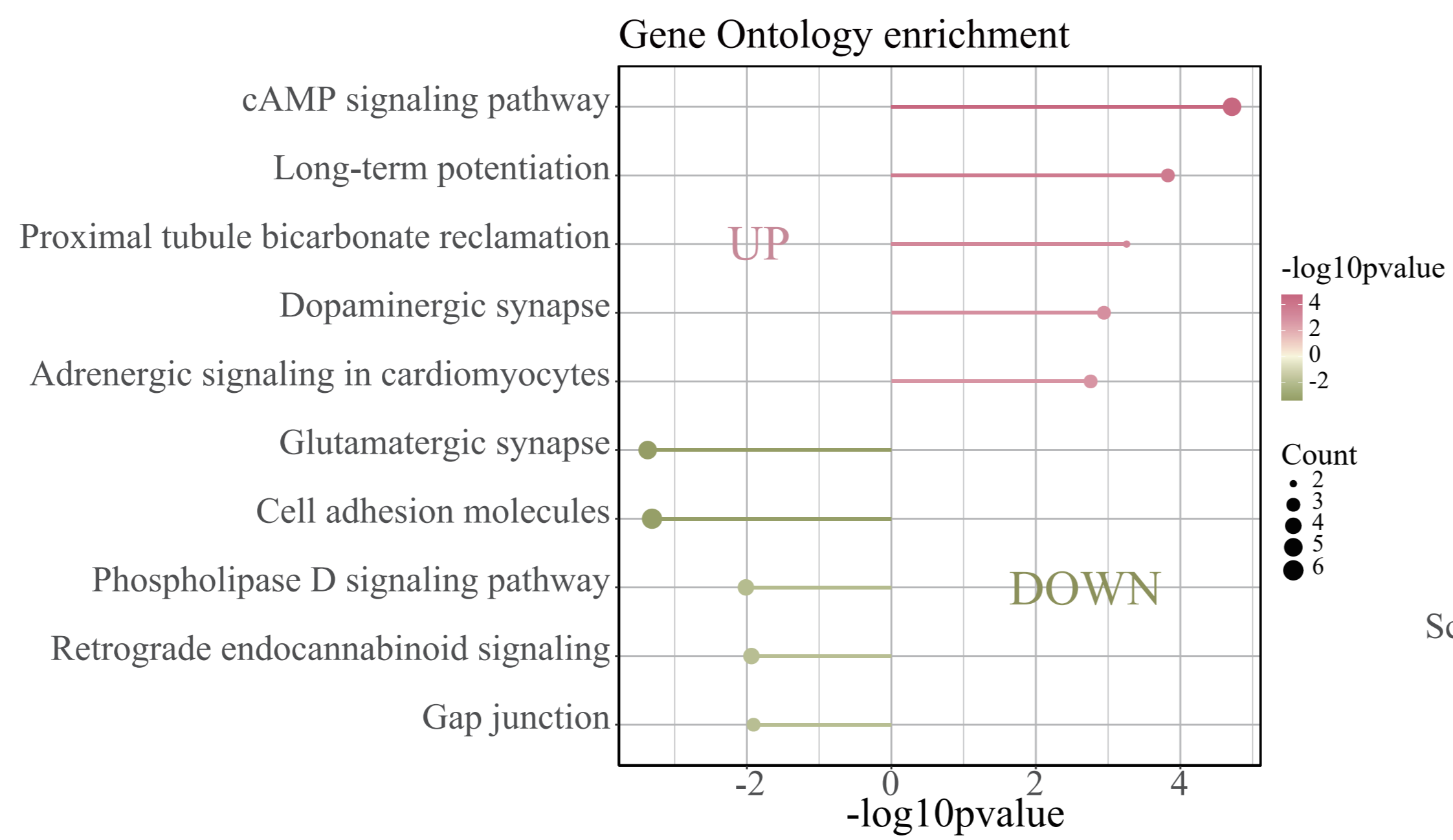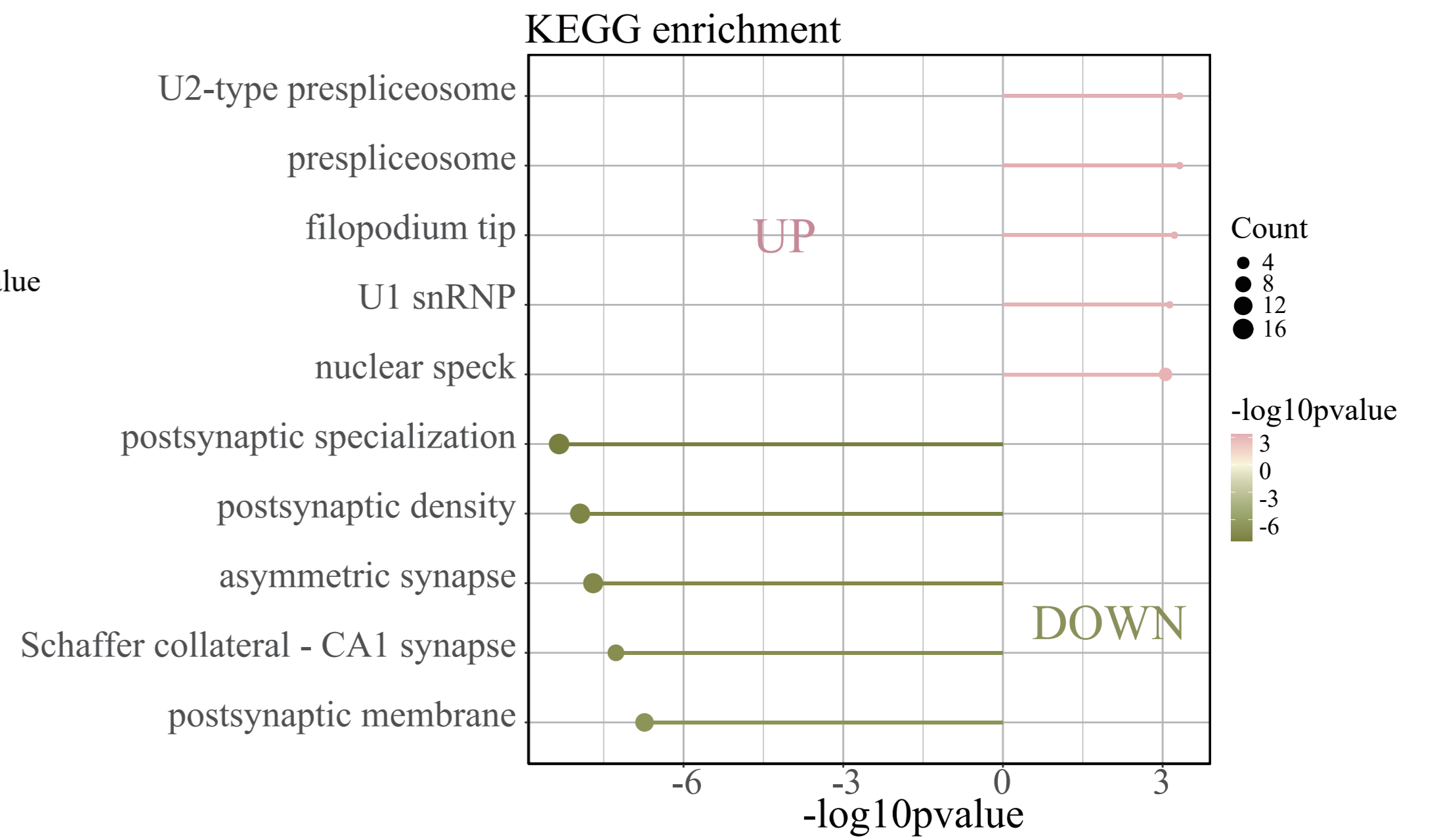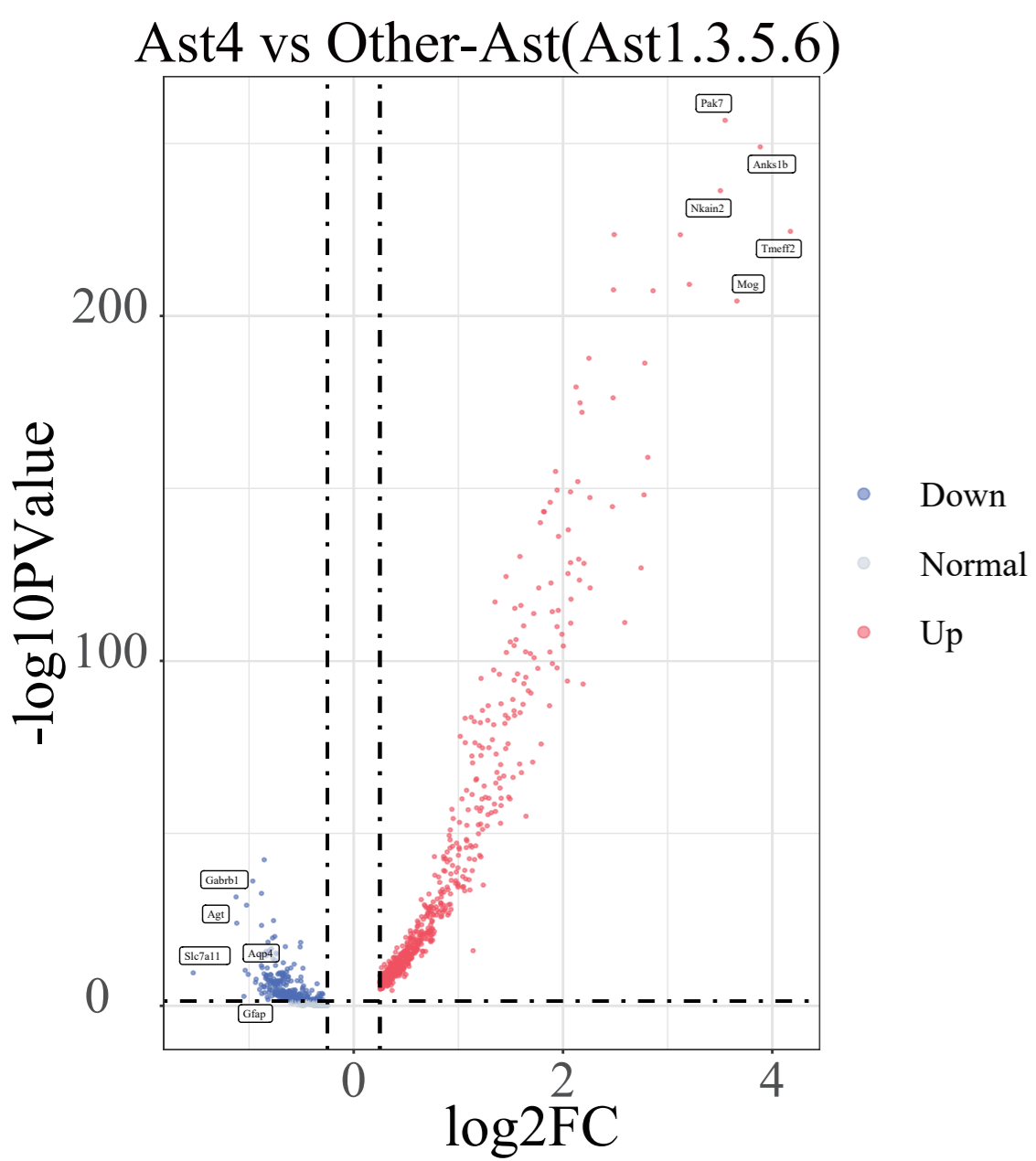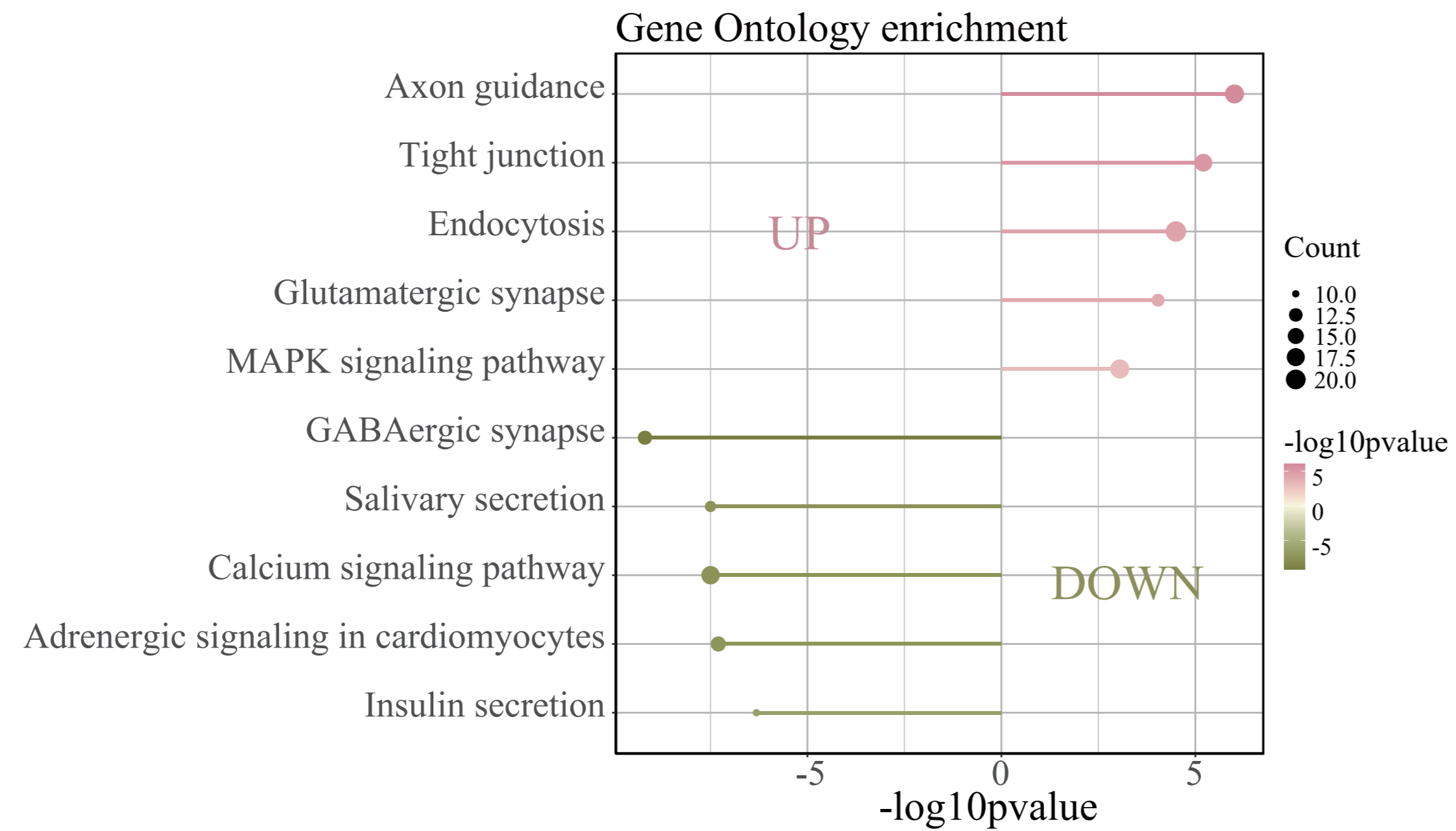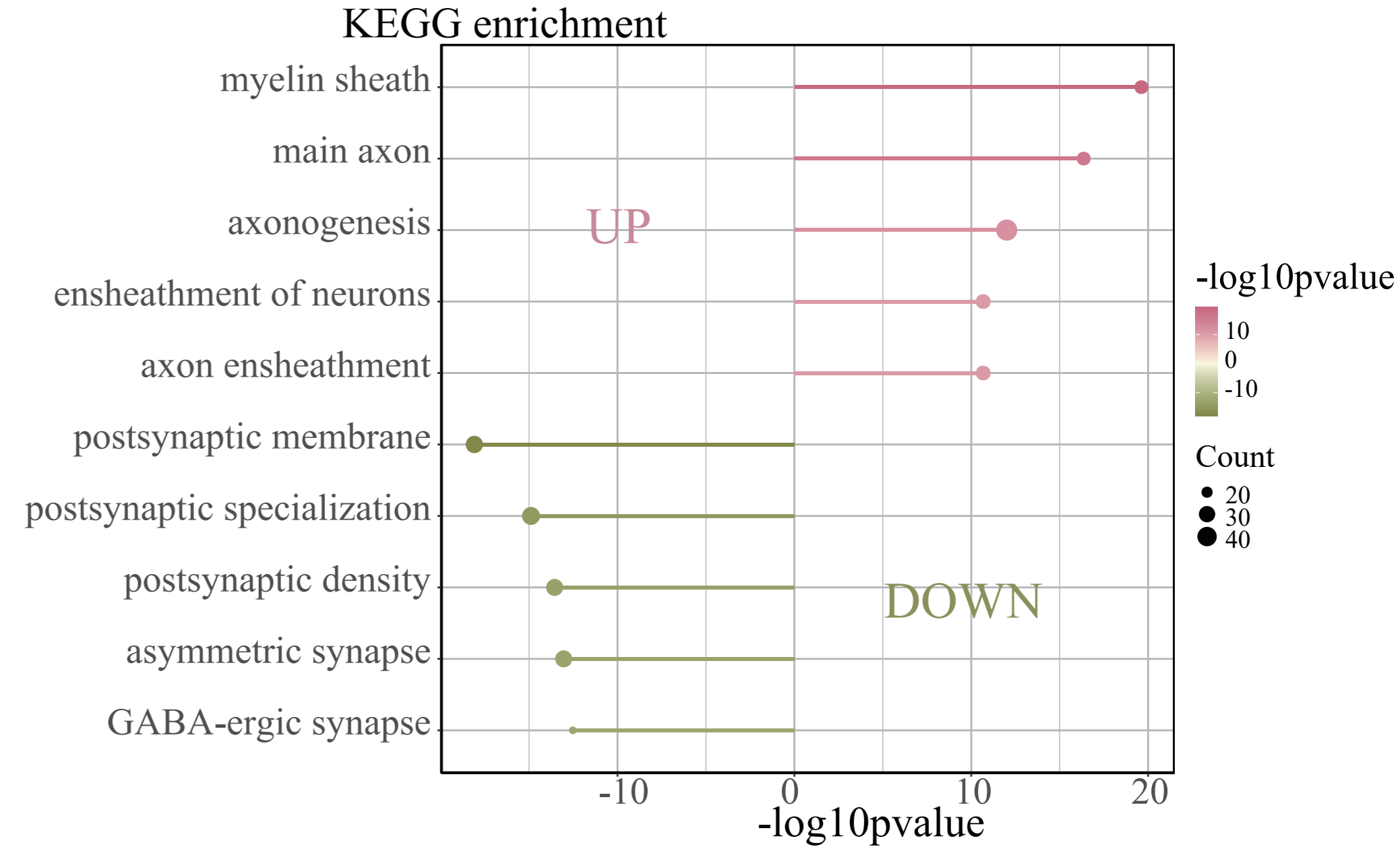

Supplement: Supplementary file 1 [file biomolecules-15-00942-s001.zip › Supplementary FIgure S3.pdf]
